# Birthweight data completeness and quality in population-based surveys: EN-INDEPTH study

**DOI:** 10.1186/s12963-020-00229-w

**Published:** 2021-02-08

**Authors:** Gashaw Andargie Biks, Hannah Blencowe, Victoria Ponce Hardy, Bisrat Misganaw Geremew, Dessie Abebaw Angaw, Alemakef Wagnew, Solomon Mekonnen Abebe, Tadesse Guadu, Justiniano S.D. Martins, Ane Baerent Fisker, Md. Ali Imam, Obed Ernest A. Nettey, Simon Kasasa, Lydia Di Stefano, Joseph Akuze, Doris Kwesiga, Joy E. Lawn, the Every Newborn-INDEPTH Study Collaborative Group, Peter Byass, Joy Lawn, Peter Waiswa, Hannah Blencowe, Judith Yargawa, Joseph Akuze, Ane B. Fisker, Justiniano S. D. Martins, Amabelia Rodrigues, Sanne M. Thysen, Gashaw Andargie Biks, Solomon Mokonnen Abebe, Tadesse Awoke Ayele, Telake Azale Bisetegn, Tadess Guadu Delele, Kassahun Alemu Gelaye, Bisrat Misganaw Geremew, Lemma Derseh Gezie, Tesfahun Melese, Mezgebu Yitayal Mengistu, Adane Kebede Tesega, Temesgen Azmeraw Yitayew, Simon Kasasa, Edward Galigawango, Collins Gyezaho, Judith Kaija, Dan Kajungu, Tryphena Nareeba, Davis Natukwatsa, Valerie Tusubira, Yeetey A. K. Enuameh, Kwaku P. Asante, Francis Dzabeng, Seeba Amenga Etego, Grace Manu, Alexander A. Manu, Obed Ernest Nettey, Sam K. Newton, Seth Owusu-Agyei, Charlotte Tawiah, Charles Zandoh, Nurul Alam, Nafisa Delwar, M. Moinuddin Haider, Md Ali Imam, Kaiser Mahmud, Angela Baschieri, Simon Cousens, Vladimir S. Gordeev, Victoria Ponce Hardy, Doris Kwesiga, Kazuyo Machiyama

**Affiliations:** 1 Dabat Research Centre Health and Demographic Surveillance System, Dabat, Ethiopia; 2 Department of Health Systems and Policy, Institute of Public Health, University of Gondar, Gondar, Ethiopia; 3 Maternal, Adolescent, Reproductive & Child Health (MARCH) Centre, London School of Hygiene & Tropical Medicine, London, UK; 4 Department of Epidemiology and Biostatistics, Institute of Public Health, University of Gondar, Gondar, Ethiopia; 5 Department of Human Nutrition, Institute of Public Health, University of Gondar, Gondar, Ethiopia; 6 Department of Environmental and Occupational Health and Safety, Institute of Public Health, University of Gondar, Gondar, Ethiopia; 7 Bandim Health Project, Bissau, Guinea-Bissau; 8 Research Centre for Vitamins and Vaccines, Statens Serum Institut, Copenhagen, Denmark; 9 Department of Clinical Research Open Patient data Explorative Network (OPEN), University of Southern Denmark, Odense, Denmark; 10 Health Systems and Population Studies Division, icddr,b, Dhaka, Bangladesh; 11 Kintampo Health Research Centre, Kintampo, Ghana; 12 IgangaMayuge Health and Demographic Surveillance System, Iganga, Uganda; 13 Makerere University Centre for Health and Population Research, Makerere, Uganda; 14 Department of Epidemiology and Biostatistics, Makerere University School of Public Health, Kampala, Uganda; 15 Faculty of Medicine, Nursing and Health Sciences, Monash University, Melbourne, Australia; 16 Dept. of Health Policy, Planning and Management, Makerere University School of Public Health, Kampala, Uganda; 17 Centre of Excellence for Maternal Newborn and Child Health Research, Makerere University, Kampala, Uganda; 18 Department of Women and Children’s Health, Uppsala University, Uppsala, Sweden

**Keywords:** Birthweight, Measurement, Household survey, Data quality, Heaping

## Abstract

**Background:**

Low birthweight (< 2500 g) is an important marker of maternal health and is associated with neonatal mortality, long-term development and chronic diseases. Household surveys remain an important source of population-based birthweight information, notably Demographic and Health Surveys (DHS) and UNICEF’s Multiple Indicator Cluster Surveys (MICS); however, data quality concerns remain. Few studies have addressed how to close these gaps in surveys.

**Methods:**

The EN-INDEPTH population-based survey of 69,176 women was undertaken in five Health and Demographic Surveillance System sites (Matlab-Bangladesh, Dabat-Ethiopia, Kintampo-Ghana, Bandim-Guinea-Bissau, IgangaMayuge-Uganda). Responses to existing DHS/MICS birthweight questions on 14,411 livebirths were analysed and estimated adjusted odds ratios (aORs) associated with reporting weighing, birthweight and heaping reported. Twenty-eight focus group discussions with women and interviewers explored barriers and enablers to reporting birthweight.

**Results:**

Almost all women provided responses to birthweight survey questions, taking on average 0.2 min to answer. Of all babies, 62.4% were weighed at birth, 53.8% reported birthweight and 21.1% provided health cards with recorded birthweight. High levels of heterogeneity were observed between sites. Home births and neonatal deaths were less likely to be weighed at birth (home births aOR 0.03(95%CI 0.02–0.03), neonatal deaths (aOR 0.19(95%CI 0.16–0.24)), and when weighed, actual birthweight was less likely to be known (aOR 0.44(95%CI 0.33–0.58), aOR 0.30(95%CI 0.22–0.41)) compared to facility births and post-neonatal survivors. Increased levels of maternal education were associated with increases in reporting weighing and knowing birthweight. Half of recorded birthweights were heaped on multiples of 500 g. Heaping was more common in IgangaMayuge (aOR 14.91(95%CI 11.37–19.55) and Dabat (aOR 14.25(95%CI 10.13–20.3) compared to Bandim. Recalled birthweights were more heaped than those recorded by card (aOR 2.59(95%CI 2.11–3.19)). A gap analysis showed large missed opportunity between facility birth and known birthweight, especially for neonatal deaths. Qualitative data suggested that knowing their baby’s weight was perceived as valuable by women in all sites, but lack of measurement and poor communication, alongside social perceptions and spiritual beliefs surrounding birthweight, impacted women’s ability to report birthweight.

**Conclusions:**

Substantial data gaps remain for birthweight data in household surveys, even amongst facility births. Improving the accuracy and recording of birthweights, and better communication with women, for example using health cards, could improve survey birthweight data availability and quality.

## Key findings

**Table:** 

**What is new?**	
• **What was known already:** Household surveys remain an important source of population-based data on birthweight, but with challenges in data quality including missing birthweights and heaping. • **What was done:** EN-INDEPTH study was a population-based survey of 69,176 women of reproductive age, including detailed assessment of questions on existing DHS/MICS questions on birthweight for 14,411 livebirths since 1 January 2012. Community perceptions of birthweight and barriers/enablers to reporting this in surveys were explored through focus group discussions with women and interviewers.	
**What was found in the quantitative data?**	
• **Completeness of data:** Overall, 62% of liveborn babies were reported to have been weighed at birth; however, weight was not known for 14%. There was marked between-site variation. In IgangaMayuge, 83% of women reported that their baby was weighed, and almost all knew the birthweight, compared to Dabat where 24% of babies were weighed, and of those, 47% of mothers did not know the birthweight. Around a third of weighed babies had a birthweight from card available at the time of the survey. Nearly 73% of birthweights in Bandim were recorded on health cards, compared to fewer than 2% in Matlab. Women took on average 0.2 min to answer direct questions on birthweight. • **Data quality:** Heaping was common and varied by site. More marked heaping was seen for recalled birthweights compared to those recorded by card. • **Data utility:** Babies who were born at home or died during the neonatal period were less likely to be weighed at birth, and when they were weighed, their birthweight was less frequently recorded on health cards or recalled by mothers at the time of the survey. • **Gap analysis** showed missed opportunities for birthweight data availability amongst facility livebirths, especially regarding availability of birthweight recorded on health cards. Birthweight data gaps were greatest for neonatal deaths.	
**What was found in the qualitative data?**	
• **Perceived value:** In all sites, birthweight was perceived as valuable by women and interviewers. Despite this, many women did not know their baby’s birthweight. • **Barriers to reporting birthweight:** o Birthweight not being measured: e.g. births in facilities without a functioning scale or when the baby was very sick, stillborn or born at home. o Birthweight not being communicated: mothers reported seeing their baby placed on scales but not being informed of the weight. Health cards are commonly used to communicate birthweight—but challenges include missing cards, cards being held by men, missing or illegible birthweight information on cards and birthweight recorded on the mother’s rather than the child’s health card. o Social perceptions and spiritual beliefs, such as fear of attacks from the ‘evil eye’, impacted negatively on both weighing and reporting of a baby’s weight in two sites (Dabat and Matlab).	
**What next in measurement and research?**	
• **Measurement improvement now:** o Improving birthweight measurement for every baby, everywhere, including those born at home, will require access to reliable, robust, low-cost scales in all facilities and relevant community settings. o Women perceive birthweight as important and should be provided with this information both verbally and in written form on health cards regardless of their baby’s outcome. o Improved interviewer training in reviewing health cards and obtaining birthweight information could improve availability of survey birthweight data. o Linking birthweight data collected outside facility-based Health Management Information Systems could increase availability of data, especially for home births and those in private sector. • **Research needed:** to understand how best to improve data quality and minimise heaping.	

## Background

Every year, around 20.5 million babies are born with a low birthweight (LBW: birthweight < 2500 g) worldwide, 80% of whom are born in low- and middle-income countries (LMICs) [1]. LBW babies are at higher risk of mortality during the neonatal and postnatal periods, plus childhood morbidity, including stunting, developmental delay, and adult-onset diabetes and heart disease [1–3]. Accurate birthweight measurement is important for the identification of individual risk (e.g. the need for extra care for small or exceptionally large infants) but also in monitoring population LBW rates and providing disaggregated data on neonatal outcomes including morbidity and mortality. Birthweight is included in the Every Newborn Action Plan (ENAP) Measurement Improvement Roadmap as a key indicator to drive improvements in global maternal and newborn health [4]. In 2012, the Global Nutrition Plan endorsed a global target of a 30% reduction in the prevalence of LBW by 2025 [5], but recent time trends and estimates suggest this target is seriously off track [1].

Birthweight is the weight of the fetus or newborn obtained immediately after birth. For livebirths, measurement of birthweight within the first hour of life before significant postnatal weight loss has occurred is preferable, although weights up to 24 h after birth are accepted [6]. Whilst routine administrative data sources such as Civil Registration and Vital Statistics (CRVS) and Health Management Information Systems (HMIS) usually collect information on birthweight, these systems often only cover a small proportion of the population in many LMICs. Facility births now account for almost 80% of all births worldwide, and some middle-income countries have made progress in the coverage and quality of routine Health Management Information Systems (HMIS), but gaps remain especially in low-income settings [7]. Therefore, most high-burden countries rely on data from household surveys to inform estimates of LBW rates [1, 8, 9].

Household surveys such as Demographic and Health Surveys (DHS) and UNICEF’s Multiple Indicator Cluster Surveys (MICS) typically collect data on birthweight using two types of questions. Firstly by collecting information on the baby’s birthweight from health cards or maternal recall, and secondly, by asking the woman her perception of her baby’s size-at-birth (very small, small, average, large or very large) [8, 9]. The latter is known to be a poor predictor of actual birthweight at the individual level [1, 10–18]. However, in view of missing birthweights for a large proportion of livebirths in some surveys who are likely to be systematically different from those reporting a weight, size-at-birth questions are retained as one of many variables to impute missing birthweights [1]. In this study, we consider only questions that sought to provide a direct measure of birthweight. Challenges with data quality for birthweight data in surveys include biases in babies with missing birthweight—these may account for a large proportion of babies in some populations and may differ systematically from those not weighed—and heaping of birthweights at 100-g or 500-g intervals in both card and recall data [1, 2, 12].

To enable high-quality data on birthweight to be available in household surveys, the baby must be accurately weighed at birth and this information communicated to the mother or caregiver (ideally recorded in both the mother’s and baby’s health cards) and accurately reported to the interviewer. There have been few systematic assessments of quality of survey measurement of birthweight to date [2, 12], or of perceived barriers and enablers both to weighing babies at birth and to reporting such information in household surveys. One of the challenges of household surveys is the length of time questionnaires take to administer. No study to date has yet explored the time taken to answer standard survey questions on birthweight.

This paper is part of a series of papers from the Every Newborn-International Network for the Demographic Evaluation of Populations and their Health (EN-INDEPTH) study in five health and demographic surveillance system (HDSS) sites in Africa and Asia. This paper addresses three objectives:

1. ***Assess the time taken to collect birthweight data***, using standard DHS/ MICS birthweight questions
2. ***Evaluate the quality of birthweight data reported in the EN-INDEPTH survey*** including missingness and heaping.
3. ***Undertake qualitative research to assess community perceptions, and barriers and enablers to reporting accurate birthweight information in household surveys*** and identify commonalities and differences across the sites

## Methods

### EN-INDEPTH study design and setting

The EN-INDEPTH study was a cross-sectional multi-site study conducted between July 2017 and August 2018, including a survey of 69,176 women aged 15–49 years in five HDSS sites: Bandim in Guinea-Bissau, Dabat in Ethiopia, IgangaMayuge in Uganda, Matlab in Bangladesh and Kintampo in Ghana (Additional file 1). The protocol is published elsewhere and provides details of site selection [19]. The primary objective of the EN-INDEPTH study was to compare two methods of retrospective recording of pregnancy outcomes in surveys: Full Birth History with additional questions on pregnancy losses (FBH+), and full pregnancy history (FPH), and these results have also been published [20].

The EN-INDEPTH study also investigated the performance of existing or modified survey questions to capture additional questions about their pregnancy and birth. These included a sub-sample of survey respondents being asked to provide answers to questions on birthweight using the three standard DHS-7/MICS questions (Table 1) for their most recent births, all neonatal deaths and stillbirths since 1 January 2012 (Additional file 2). This paper presents findings for livebirths only. Findings for stillbirths including birthweight are presented elsewhere [21].

**Table 1:** EN-INDEPTH survey questions for birthweight

Standard questions in DHS-7 and MICS6 and EN-INDEPTH survey^a^		
Question	Details	Included in analyses presented in this paper
‘When NAME was born, was this baby^b^ very large, larger than average, average, smaller than average, or very small?’	Acceptable responses ‘very large’, ‘larger than average’, ‘average’, ‘smaller than average’, ‘very small’ or ‘don’t know’.	No^c^
‘Was NAME weighed at birth?’	Responses coded – Yes/ No/ Don’t Know.	Yes
‘How much did NAME weigh?’	Coded ‘from card’ or ‘from recall’. Response – numeric integer. Acceptable range 0.3 – 6.5kg. Red error message prompting interviewer to correct entry if value outside this range entered.	Yes

^a^These questions were asked in the EN-INDEPTH survey for all last neonatal deaths and stillbirths since 1st Jan 2012 and a subset of surviving livebirths

^b^Note very minor variation between surveys: DHS-7 repeats NAME here, MICS6 says ‘he/she’; EN-INDEPTH ‘this baby’

^c^Questions regarding maternal perception of size were excluded from these analyses as they do not provide a direct measure of birthweight

Both woman and interviewer data were collected on Android tablets using the Survey Solutions data collection and management system [22]. Interviewers were recruited locally and were familiar with the culture and dialect of the study area. Following completion of data collection, data from the five HDSS sites were anonymised by local HDSS scientists, encrypted and then shared [19]. Data management and analysis were done using Stata version 15.1.

Focus group discussions (FGDs) with survey respondents and interviewers, and a survey of interviewers, were performed between February and August 2018 [23]. Information on perceptions, practices and barriers relating to knowledge and reporting of birthweight was collected. Qualitative data were transcribed using a combination of notes and audio recordings and were coded and analysed using NViVo 12.

Results are reported in accordance with STROBE Statement checklists for cross-sectional studies [24] (Additional file 3).

### Methods by objective

#### Objective 1: assess the time taken to collect birthweight data

Time taken to complete each specific question was assessed using the EN-INDEPTH survey paradata. Paradata are collected by the app during the survey to provide detailed records of the data entry and corrections for each question, stored as time-stamped ‘events’. Time taken for each question was defined as the time interval between the time-stamp for the question(s) under study and the previous question.

#### Objective 2: evaluate the quality of reported birthweight data

Sample weights were applied in all analyses using the *svyset* command to account for the different probability of a neonatal death being included compared to a livebirth surviving the neonatal period (post-neonatal survivor), given that women’s response may vary for these two groups (Additional file 4).

Reported birthweight data were assessed for completeness, evidence of heaping on multiples of 500 g and plausibility of birthweight distribution, including mean birthweight and prevalence of LBW comparing post-neonatal survivors to neonatal deaths by women and child characteristics using descriptive statistics. We used logistic regression to identify factors associated with women’s report of birthweight information, including being weighed at birth, being able to provide a birthweight and a card birthweight, and reporting a birthweight heaped on multiples of 500 g. A data gap analysis for birthweight by site was undertaken to assess gaps in coverage of birthweight data.

#### Objective 3: assess community perceptions and barriers and enablers to reporting accurate birthweight information in household surveys

To contribute to the understanding of the measurement of birthweight in population-based surveys, FGDs were undertaken with survey respondents, interviewers and supervisors (see Additional file 5) [23]. To identify community perceptions, practices and barriers to reporting birthweight, thematic analysis was conducted using an iterative process guided by an a priori codebook and addition of new codes that emerged during analysis [23]. Themes were summarised and grouped to explore how findings contribute to understanding of the measurement of birthweight in population-based surveys.

## Results

### Overall

**Fig. 1:**
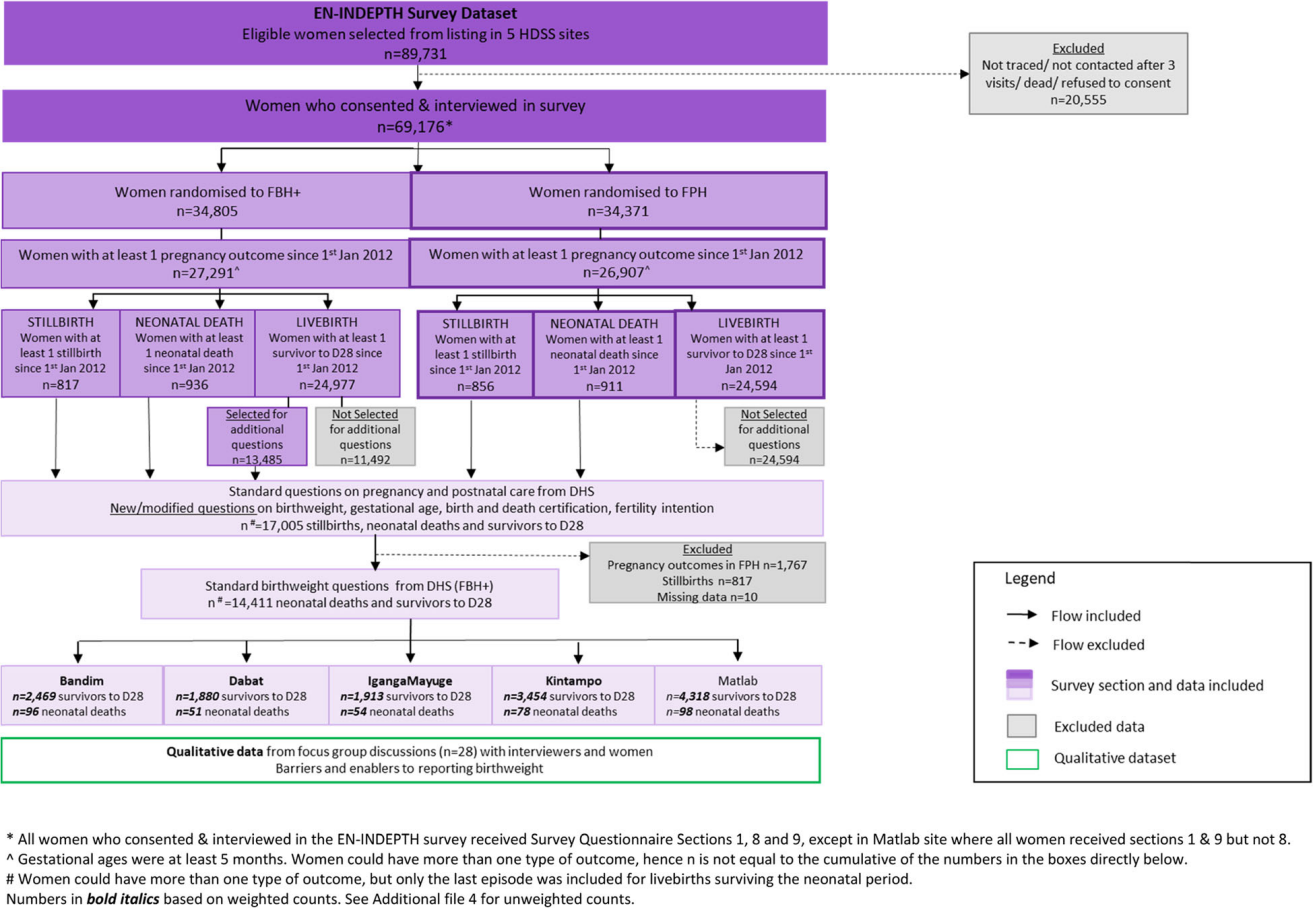
Flow diagram of EN-INDEPTH study population showing data included for birthweight analyses

Information on birthweight was analysed for 14,411 livebirths including 377 neonatal deaths since 1 January 2012 (Fig. 1 and Additional file 4). Weighted counts are presented throughout. A total of 28 FGDs were conducted, of which 19 were conducted with women (*n* = 172) and nine with survey interviewers (*n* = 82).

### Objective 1: assess the time taken to collect birthweight data

Overall, 100% of women provided valid responses to the birthweight questions. Nearly all women were able to categorise their baby by perceived size at birth, with fewer than 1% responding ‘don’t know’. Four percent of mothers were unsure if their baby was weighed at birth. For post-neonatal survivors, the perceived size questions took on average 0.4 min to complete, with some variation by site (ranging from an average of 0.2 min in Dabat to 0.7 min in Bandim), the average time was slightly longer for neonatal deaths (0.5 min) (Additional files 6.1 - 6.3). Questions regarding reported birthweight took on average 0.2 min to complete for post-neonatal survivors with some variation by site (ranging from an average of 0.1–0.2 min). Birthweights from card took on average 0.2 min to record, compared to 0.1 min for recalled birthweights (*p* < 0.001).

### Objective 2: evaluate the quality of reported birthweight data

Survey respondents differed across HDSS with regard to age, education, facility birth rates and religion (Additional file 7.1). Overall, 62.4% of women reported that their baby was weighed at birth; however, of these, 13.7% did not know the weight (Tables 2, 3, and 4). Babies who had died in the neonatal period were less likely to have been weighed at birth (aOR 0.19 (95%CI 0.16–0.24)), and if weighed, their mothers were less likely to be able to report the weight compared to women with surviving babies (aOR 0.30(95%CI 0.22–0.41)) (Additional files 7.2, 7.3). Home births were less likely to be reported as weighed at birth compared to facility births (aOR 0.03 (95%CI 0.02–0.03)) and if weighed were less likely to have a reported birthweight (aOR 0.44(95%CI 0.33–0.58)).

**Table 2:** Summary of reporting of birthweight for livebirths, EN-INDEPTH survey (*n* = 14,411)

	Overall number of babies included	Mother reported baby weighed *n* (%)	Birthweight not known by mother *n* (%)	Overall mean birthweight (kg) (95%CI)	Low birthweight overall *n* (%)
**Overall**	14411	8993 (62.4)	1233 (13.7)	3.06 (3.04–3.08)	1076 (13.9)
**Child’s characteristics**					
**Sex**					
Female	7161	4443 (62.1)	612 (13.8)	3.00 (2.97–3.02)	567 (14.8)
Male	7265	4550 (62.8)	622 (13.7)	3.12 (3.09–3.15)	509 (13.0)
**Vital status**					
Neonatal death	376	158 (42.1)	41 (25.9)	2.88 (2.75–3.01)	36 (30.6)
Post-neonatal survivor	14030	8835 (63.0)	1192 (13.5)	3.06 (3.04–3.08)	1040 (13.6)
**Site**					
Bandim	2560	1775 (69.3)	141 (8.0)	3.20 (3.15–3.25)	195 (11.9)
Dabat	1931	460 (23.8)	215 (46.8)	3.07 (3.00–3.14)	21 (8.5)
IgangaMayuge	1968	1634 (83.1)	13 (0.8)	3.24 (3.19–3.28)	16 (9.9)
Matlab	4416	3042 (68.9)	307 (10.1)	2.83 (2.80–2.87)	600 (22.0)
Kintampo	3532	2082 (59.0)	557 (26.7)	3.11 (3.08–3.15)	100 (6.6)
**Place of birth**					
Facility	8756	7451 (85.1)	965 (13.0)	3.10 (3.07–3.12)	764 (11.8)
Home	4526	560 (12.4)	146 (26.1)	3.03 (2.92 – 3.15)	74 (17.9)
Unknown	964	870 (90.3)	117 (13.4)	2.77 (2.71–2.83)	216 (28.7)
**Time from birth to survey**					
< 1 year	4025	2589 (64.3)	249 (9.6)	3.10 (3.06–3.14)	245 (10.4)
1–< 2 years	3610	2291 (63.5)	291 (12.7)	3.08 (3.04–3.12)	276 (13.8)
2–< 3 years	2759	1709 (61.9)	267 (15.6)	3.03 (2.98–3.07)	215 (14.9)
3–< 4 years	1972	1247 (63.2)	211 (16.9)	3.01 (2.96–3.06)	169 (16.3)
4–< 5 years	1273	759 (59.6)	128 (16.8)	3.02 (2.96–3.09)	112 (17.7)
5 or more years	767	398 (51.9)	88 (22.1)	2.98 (2.90–3.08)	60 (19.3)
**Mother’s characteristics**					
**Education**					
None	3958	1540 (38.9)	423 (27.5)	3.11 (3.07–3.17)	133 (11.9)
Primary	4509	2886 (64.01)	440 (15.2)	3.11 (3.07–3.14)	301 (12.3)
Secondary	4930	3682 (74.7)	326 (8.9)	2.99 (2.96–3.02)	554 (16.5)
Higher	1010	886 (87.8)	44 (5.0)	3.11 (3.06–3.16)	88 (10.5)
**Socioeconomic status**					
1 (poorest)	3149	1618 (51.4)	224 (13.8)	3.04 (2.99–3.09)	221 (15.9)
2	2884	1701 (59.0)	217 (12.8)	3.04 (2.99–3.08)	242 (16.3)
3	2813	1704 (60.6)	238 (14.0)	3.02 (2.98–3.06)	223 (15.2)
4	2839	1952 (68.8)	282 (14.4)	3.07 (3.03–3.11)	219 (13.1)
5 (richest)	2722	2018 (74.1)	273 (13.5)	3.11 (3.07–3.15)	170 (9.7)
**Parity**					
1	2413	1833 (73.6)	187 (10.6)	2.96 (2.92–2.99)	264 (16.6)
2	3653	2545 (68.1)	288 (11.6)	2.99 (2.95–3.02)	338 (15.4)
3	2734	1732 (62.5)	226 (13.3)	3.04 (3.00–3.09)	214 (14.5)
4	1798	1061 (58.4)	162 (15.4)	3.13 (3.07–3.19)	102 (11.5)
5+	3808	1972 (51.8)	370 (18.8)	3.23 (3.18–3.28)	158 (9.9)
**Age**					
< 20	504	335 (66.4)	26 (7.7)	2.91 (2.82–3.00)	55 (17.9)
20–24	3090	1992 (64.5)	229 (11.5)	3.00 (2.96–3.04)	260 (14.8)
25–29	4024	2596 (64.5)	335 (12.9)	3.01 (2.97–3.05)	358 (15.8)
30–34	3182	2017 (63.4)	251 (12.4)	3.12 (3.08–3.16)	219 (12.4)
35+	3605	2053 (57.0)	393 (19.2)	3.15 (3.11–3.19)	184 (11.1)

Rounded numbers based on weighted counts presented and may not sum to 14,411

**Table 3:** Comparison of reporting of birthweight for livebirths by card and recall, EN-INDEPTH survey (*n* = 7760)

	Birthweight from recall			Birthweight from card		
	Number of babies	Mean birthweight (kg) (95% CI)	Low birthweight *n* (%)	Number of babies	Mean birthweight (kg) (95% CI)	Low birthweight *n* (%)
**Overall**	4702	3.01 (2.98–3.04)	802 (17.1)	3057	3.13 (3.10–3.16)	274 (9.0)
**Child’s characteristics**						
**Sex**						
Female	2329	2.96 (2.92–2.99)	420 (18.0)	1503	3.06 (3.02–3.09)	147 (9.8)
Male	2374	3.06 (3.03–3.10)	382 (16.1)	1555	3.20 (3.16–3.24)	128 (8.2)
**Vital status**						
Neonatal death	102	2.88 (2.74–3.03)	31 (30.1)	15	2.82 (2.48–3.16)	5 (34.2)
Post-neonatal survivor	4600	3.01 (2.99–3.04)	771 (16.8)	3042	3.13 (3.10–3.16)	269 (8.9)
**Site**						
Bandim	441	3.29 (3.18–3.40)	70 (15.9)	1193	3.17 (3.12–3.22)	129 (10.6)
Dabat	215	3.08 (3.01–3.16)	18 (8.4)	29	2.97 (2.80–3.15)	3 (8.9)
IgangaMayuge	1165	3.25 (3.20–3.31)	114 (9.8)	456	3.20 (3.12–3.27)	45 (9.8)
Matlab	2692	2.84 (2.80–2.87)	589 (21.9)	44	2.71 (2.51–2.90)	12 (26.1)
Kintampo	189	3.28 (3.19–3.37)	10 (5.5)	1336	3.09 (3.06–3.12)	92 (6.8)
**Mother’s characteristics**						
**Education**						
No education	315	3.13 (3.00–3.25)	62 (19.6)	802	3.12 (3.06–3.17)	76 (9.4)
Primary	1249	3.08 (3.02–3.14)	204 (16.3)	1197	3.13 (3.09–3.18)	97 (8.0)
Secondary	2445	2.94 (2.91–2.98)	461 (18.9)	910	3.12 (3.07–3.16)	94 (10.2)
Higher	693	3.08 (3.02–3.14)	75 (10.8)	149	3.26 (3.13–3.38)	14 (8.9)
**Socioeconomic status**						
1 (poorest)	771	2.95 (2.87–3.02)	165 (21.4)	623	3.16 (3.10–3.22)	57 (9.1)
2	823	2.96 (2.90–3.03)	179 (21.8)	661	3.13 (3.06–3.19)	63 (9.6)
3	867	2.98 (2.92–3.04)	151 (17.4)	600	3.08 (3.02–3.15)	73 (12.1)
4	1051	3.04 (2.98–3.10)	167 (15.9)	619	3.12 (3.07–3.18)	52 (8.4)
5 (richest)	1191	3.09 (3.04–3.13)	140 (11.8)	554	3.16 (3.10–3.22)	30 (5.3)
**Parity**						
1	1092	2.94 (2.90–2.99)	198 (18.1)	497	2.99 (2.94–3.04)	66 (13.3)
2	1521	2.93 (2.88–2.97)	286 (18.8)	680	3.11 (3.06–3.17)	52 (7.6)
3	932	2.96 (2.90–3.03)	175 (18.7)	549	3.18 (3.11–3.25)	40 (7.2)
4	434	3.16 (3.06–3.26)	54 (12.5)	454	3.11 (3.03–3.18)	48 (10.6)
5+	723	3.26 (3.19–3.33)	89 (12.4)	878	3.20 (3.14–3.26)	69 (7.9)
**Age**						
< 20	204	2.92 (2.80–3.03)	39 (19.1)	105	2.90 (2.76–3.03)	16 (15.6)
20–24	1184	2.98 (2.93–3.03)	197 (16.7)	580	3.04 (2.98–3.10)	63 (10.9)
25–29	1477	2.95 (2.90–2.99)	285 (19.3)	784	3.13 (3.07–3.18)	72 (9.2)
30–34	1003	3.09 (3.04–3.15)	155 (15.4)	763	3.15 (3.09–3.22)	64 (8.4)
35+	834	3.09 (3.03–3.15)	126 (15.1)	826	3.20 (3.16–3.25)	59 (7.1)

Rounded numbers based on weighted counts presented and may not sum to 7760

**Table 4:** Comparison of reporting of birthweight for neonatal deaths and surviving livebirths, EN-INDEPTH survey (*n* = 7760)

	Neonatal deaths			Post-neonatal survivor		
	Number of babies	Mean birthweight (kg) (95% CI)	Low birthweight *n* (%)	Number of babies	Mean birthweight (kg) (95% CI)	Low birthweight *n* (%)
**Overall**	117	2.88 (2.75–3.01)	36 (30.6)	7643	3.06 (3.04–3.08)	1040 (13.6)
**Child’s characteristics**						
**Sex**						
Female	43	2.86 (2.65–3.07)	13 (30.8)	3789	3.00 (2.97–3.02)	554 (14.6)
Male	74	2.89 (2.72–3.06)	23 (30.5)	3854	3.12 (3.09–3.15)	487 (12.6)
**Site**						
Bandim	35	3.24 (3.06–3.45)	5 (15.8)	1599	3.20 (3.15–3.25)	186 (11.8)
Dabat	3	3.44 (2.69–4.19)	0 (11.1)	241	3.06 (2.99–3.13)	20 (8.4)
IgangaMayuge	23	3.15 (2.89–3.42)	5 (21.0)	1598	3.24 (3.19–3.28)	155 (9.7)
Matlab	49	2.48 (2.28–2.68)	22 (45.2)	2686	2.84 (2.81–2.87)	578 (21.5)
Kintampo	7	2.63 (2.08–3.18)	3 (42.1)	1519	3.12 (3.08–3.15)	98 (6.4)
**Mother’s characteristics**						
**Education**						
None	15	3.13 (2.76–3.50)	3 (22.5)	1102	3.11 (3.06–3.17)	130 (11.8)
Primary	36	2.99 (2.75–3.23)	10 (28.3)	2410	3.10 (3.07–3.15)	291 (12.1)
Secondary	51	2.77 (2.59–2.95)	17 (33.6)	3304	2.99 (2.96–3.02)	537 (16.2)
Higher	15	2.73 (2.30–3.12)	5 (34.2)	827	3.12 (3.07–3.17)	83 (10.0)
**Socioeconomic status**						
1 (poorest)	23	2.99 (2.79–3.19)	6 (25.0)	1371	3.04 (2.99–3.09)	215 (15.7)
2	21	2.77 (2.48–3.06)	9 (41.4)	1463	3.04 (2.99–3.09)	234 (16.0)
3	25	2.70 (2.38–3.02)	10 (38.2)	1441	3.03 (2.98–3.07)	214 (14.8)
4	20	3.05 (2.72–3.38)	4 (22.2)	1650	3.07 (3.03–3.11)	215 (13.0)
5 (richest)	28	2.90 (2.61–3.19)	7 (26.3)	1717	3.11 (3.07–3.15)	163 (9.5)
**Parity**						
1	14	2.79 (2.47–3.11)	4 (32.4)	1575	2.96 (2.93–2.99)	259 (16.5)
2	36	2.91 (2.71–3.10)	10 (27.3)	2165	2.98 (2.95–3.02)	328 (15.2)
3	22	2.68 (2.34–3.06)	8 (35.6)	1459	3.05 (3.00–3.10)	206 (14.1)
4	17	2.75 (2.40–3.10)	7 (40.4)	871	3.14 (3.08–3.20)	95 (10.9)
5+	29	3.10 (2.81–3.40)	7 (24.4)	1572	3.24 (3.19–3.29)	152 (9.6)
**Age**						
< 20	4	2.35 (1.79–2.91)	2 (50.0)	305	2.91 (2.83–3.01)	53 (17.4)
20–24	31	2.77 (2.50–3.04)	11 (34.5)	1732	3.00 (2.96–3.04)	250 (14.4)
25–29	28	2.80 (2.55–3.05)	9 (31.2)	2233	3.01 (2.97–3.04)	349 (15.6)
30–34	25	3.11 (2.83–3.38)	7 (26.1)	1741	3.12 (3.08–3.16)	212 (12.2)
35+	29	2.95 (2.68–3.21)	8 (26.9)	1631	3.15 (3.11–3.20)	177 (10.8)

Rounded numbers are based on weighted counts presented and may not sum to 7760. Calculations are based on unrounded numbers

Just over half of women in the poorest quintile reported that their baby was weighed whilst this was three quarters of the wealthiest (Table 2). After adjusting for other factors, women in the wealthiest quintile were more likely to report that their baby was weighed compared to those in the poorest (aOR 1.53(95%CI 1.24–1.86)); they were more likely to be able to provide a birthweight (aOR 1.48(95%CI 1.13–1.95)), but the birthweight was less likely to be from a card (aOR 0.59(95%CI 0.48–0.73)) (Additional files 7.2, 7.3).

Educated women were more likely to report that their baby was weighed compared to women with no education (Primary: aOR 1.43(95%CI 1.20–1.71); Secondary: aOR 2.12(95%CI 1.73–2.61); Higher: aOR 3.92(95%CI 2.96–5.18)). When their baby was weighed, they were more likely to be able to report the actual birthweight (Primary: aOR 1.30(95%CI 1.05–1.62); Secondary: aOR 2.07(95%CI 1.60–2.67); Higher: aOR 5.25(95%CI 3.27–8.43)). There was large variation between sites with over 80% of women in IgangaMayuge reporting that their baby was weighed, and almost all able to provide the birthweight, compared with Dabat, where fewer than a quarter of babies were weighed and in half of those weighed the mothers did not know the birthweight (Table 2).

Birthweight was available from card for 21.2% of all babies, with a further 32.6% having birthweight from recall (Table 3). Babies from the Bandim site were more likely to have a card birthweight than babies in all other sites (aOR 6.51(95%CI 5.50–7.70)). Cards completed with a birthweight were less likely to be available for home births compared to facility births (aOR 0.06(95%CI 0.05–0.08)) and neonatal deaths compared to babies surviving the neonatal period (aOR 0.07 (95%CI 0.05–0.10)) (Additional file 7.4). Mean birthweight reported by card was higher than from recall (*p* < 0.001). However, excluding births in Matlab where mean birthweight was substantially lower than in the other sites and birthweight by card was rarely available, mean birthweight from recall was slightly higher than that from card (3.24 kg compared to 3.14 kg (*p* < 0.001) (Additional file 7.5).

Overall, neonatal deaths with a known birthweight had a lower mean birthweight compared to post-neonatal survivors (2.88 kg vs 3.06 kg, *p* = 0.006) (Table 4). As expected, the proportion of neonatal deaths that were LBW was much higher than the proportion LBW amongst babies surviving the neonatal period (30.6% compared to 13.6%, *p* < 0.001).

**Fig. 2:**
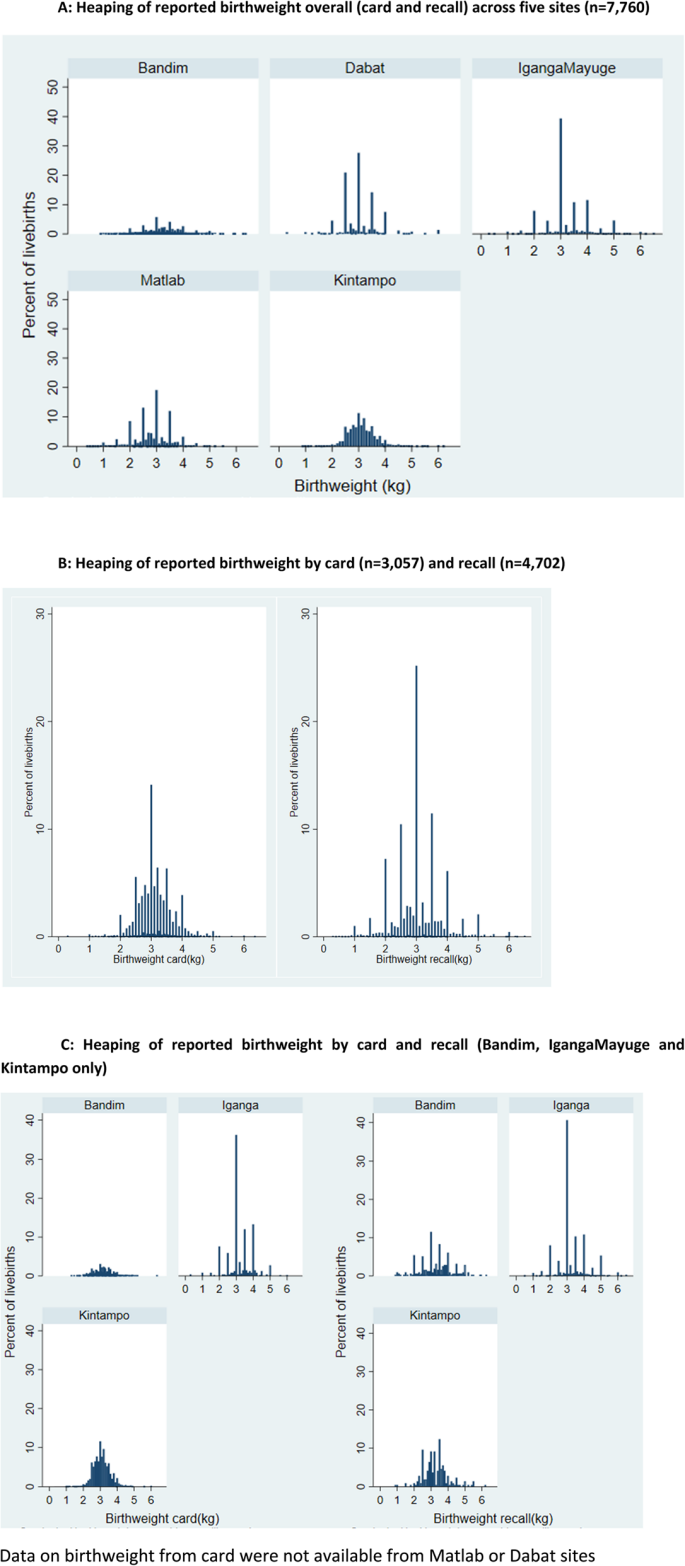
Heaping of reported birthweight by site and data source

Consistent with previous studies, heaping was common, with 50.7% of all reported birthweights heaped on multiples of 500 g. Heaping varied by site, with marked heaping in 3 out of 5 sites (Dabat, IgangaMayuge and Matlab) (Fig. 2a and Table 5). Heaping was more marked for recalled birthweights compared to those from card (aOR 2.59(95%CI 2.11–3.19)) (Fig. 2b and Table 5). After adjusting for other factors, heaping on 500 g was not associated with socioeconomic status, but was less common in women with secondary or higher education compared to those with primary only or no education (Additional file 7.6). Heaping was most marked in IgangaMayuge, where around 80% of birthweights were heaped on multiples of 500 g (78.4% for card and 82.1% for recalled birthweights) (Fig. 2c).

**Table 5:** Heaping indices for birthweight, overall and by card and recall (*n* = 7760)

	Bandim (Guinea-Bissau)		Dabat (Ethiopia)		IgangaMayuge (Uganda)		Matlab (Bangladesh)		Kintampo (Ghana)		Overall	
	Card	Recall	Card	Recall	Card	Recall	Card	Recall	Card	Recall	Card	Recall
1000 g	0.0	0.8	1.0	2.0	4.0	9.0	0.0	1.5	0.3	1.0	1.5	1.8
1500 g	0.0	1.5	0.0	2.0	4.0	15.0	1.1	1.4	0.2	2.0	0.5	1.9
2000 g	0.1	1.6	2.0	10.5	18.0	18.6	4.0	1.9	0.3	0.4	1.0	2.8
2500 g	0.1	0.8	8.0	3.6	3.1	2.3	1.0	1.3	0.4	1.3	0.4	1.6
3000 g	0.1	1.1	2.0	4.6	5.9	8.3	1.2	1.7	0.4	0.4	0.6	2.7
3500 g	0.1	0.6	3.3	3.3	2.6	3.3	0.8	2.0	0.4	0.8	0.4	1.9
4000 g	0.1	0.7	3.0	11.7	3.0	3.9	0.0	1.3	0.4	0.4	0.3	2.0

Calculated as: number of babies with a given birthweight / number of babies with a birthweight within 249 g above or below the given birthweight, e.g. number exactly on 1500 g / (number (1251 g to 1499 g) + number (1501 g to 1749 g))

**Fig. 3:**
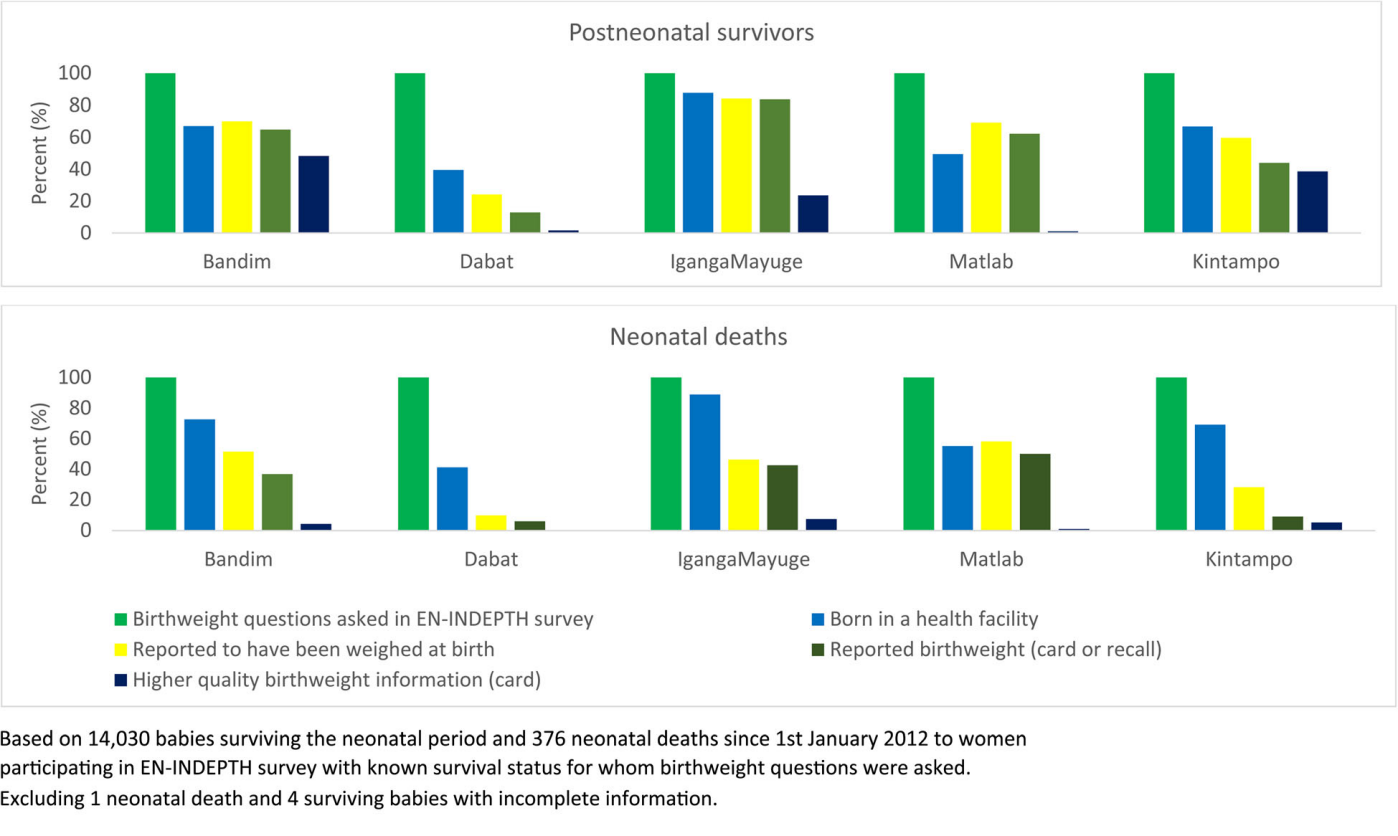
Data gap analysis for birthweight for neonatal deaths and post-neonatal survivors, by site (*n* = 14,406)

In Kintampo and Dabat, there remains a large gap between facility births and mother’s knowledge of birthweight, especially for neonatal deaths (Fig. 3). In Matlab and Bandim, a slightly higher proportion of all babies were weighed than the proportion who were born in facilities, suggesting that some weighing is being undertaken at the community level or that some women are able to access healthcare after a home birth in these settings. Availability of birthweight data from card was low in Dabat, IgangaMayuge and Matlab, compared to the proportion of babies that were reported to have been weighed in a facility.

### Objective 3: assess community perceptions and barriers and enablers to reporting accurate birthweight information in household surveys

#### Community perceptions

In all sites knowing birthweight was perceived by women to be valuable for livebirths. Women reported that it was useful to assess whether the baby was healthy, to guide care of babies identified as small and to provide a baseline for future growth monitoring (Additional file 8.1).

#### Reported barriers and enablers

Home birth was a barrier to birthweight measurement in all sites, with community-based healthcare workers and traditional birth attendants reported to rarely have a weighing equipment. In Bandim and Kintampo, barriers to taking a newborn born at home to a health facility to be weighed included concerns that the health staff would not provide care, such as weighing the baby. In Dabat, women were concerned about exposing a baby to sun and wind when being weighed.

*“At home it [measuring birthweight] is rare. In the hospitals the doctors themselves take the weight”* (Woman, Matlab, Bangladesh).

*“It is good if you give birth in the hospital to weigh the child, but I gave birth at home and my baby was not weighed, only when I went to look for a card was he weighed”* (Woman, Bandim, Guinea-Bissau).

Other reported barriers included lack of functioning weighing scales in some health facilities, especially private ones. In general, most participants across all sites did not perceive any value in weighing dead babies, whether stillborn or dying soon after birth. Additionally, in Matlab and Dabat, there was concern that knowing the weight of a baby could be associated with attacks from the ‘evil eye’, leading to harm and possible death.

*“I had lost child after his weight has been taken by the community data collector”* (Woman, Dabat, Ethiopia).

A lack of knowledge of their baby’s birthweight was an important barrier to women reporting this information in the survey in all sites (Additional file 8.2). Barriers to women knowing their babies’ birthweight included separation of mother and baby soon after birth, for example with very sick or stillborn babies, women not being told their babies’ birthweight even when the baby was weighed, and women finding it difficult to ask healthcare workers for the information.

*“The relationship between the women and the health care providers also serves as a factor, sometimes they do not ask questions …..there should be a system whereby these women can ask questions about their health and that of their child. [For] example what the weight of their baby was at birth”* (Data collector, Kintampo, Ghana).

Interviewers considered educated women more able to provide information on the birthweight of their child. In addition, health cards were important enablers to reporting birthweight information in the survey. However, many barriers to the effectiveness of health cards were found. These included cards misplaced or held by the men in the family, cards with illegible or missing birthweight entries, or the wrong card being shown to the interviewer, for example when birthweight was recorded in the antenatal card but not transcribed to the child’s health card. Importantly for women who were illiterate, they may value the card but not have access to the information.

*“I went ahead to ask ‘was the baby weighed at birth?’ and she told me yes and then I asked her what was the weight of the baby and she told me ‘I don’t know and that is why I have given you the card so that I may also know’”* (Data collector, IgangaMayuge, Uganda).

## Discussion

Information on birthweight has been collected in standard DHS surveys since 1988 (Phase 2 onwards). Previous studies have questioned the quality of birthweight data from such surveys, and methods have been developed to enable their use to produce estimates of LBW based on available data [1, 2]. However, few studies have looked in detail, combining quantitative and qualitative analyses, at the time taken for women to answer these questions and the factors associated with missing and low-quality birthweight data in order to inform birthweight data improvements in surveys. Birthweight questions took less than 1 min on average to answer. We found gaps in availability of birthweight data particularly for home births and neonatal deaths, as well as major issues of heaping for birthweight data reported from both card and recall—up to 80% of data being heaped in some sites. In addition, in this study, we address an important previously identified gap in the understanding of community and family demand for birthweight data and found that knowing birthweight was universally perceived as important for livebirths across these five different populations in Asia and Africa. Improving birthweight data in surveys is likely to require improvements in measurement of birthweight at the time of birth, improved communication between health workers and women and further improvements in survey questions and implementation.

Home birth remains a large barrier to measuring birthweight. We found that just 12.4% of women with home births reported that their baby was weighed, compared to 85.1% of facility births. In view of the barriers of accessing facilities in the immediate postnatal period, interventions to increase birthweight measurement should focus on bringing accurate weighing equipment to the mothers and newborn. This will require improved, low-cost, robust and accurate weighing scales to be made available in communities. There is limited literature on potential innovations to improve the measurement of birthweight for home births, although the provision of weighing scales, training of healthcare workers and traditional birth attendants, and community engagement have been shown to increase coverage of weighing at birth [25–27]. The person best placed to weigh babies at birth in the community is likely to vary in different settings and may include community volunteers, community midwives or traditional birth attendants. The findings of this study highlight the importance of understanding local context-specific barriers which may impact successful implementation of community weighing, for example fear of witchcraft or the ‘evil eye’ and concerns about exposing the newborn to the elements.

Coverage of facility births is increasing in all settings [28], and the weighing of newborns should be relatively straightforward for these births [7, 29]. However, in this study women reported lack of suitable weighing scales in health facilities, especially in the private sector. The finding of the lack of birthweight measurement for very sick or stillborn babies is similar to other studies [1]. However, this finding may be context-specific. For example, the Every Newborn-BIRTH multi-site study found that the majority of stillbirths were routinely weighed in four out of five study facilities, but that weight was not always communicated to the mothers [30].

In many settings, information about birthweight is communicated verbally to the woman and her family soon after the baby is weighed. However, high workload and time pressures on healthcare workers, and healthcare workers’ perceptions of a woman’s desire or need for this information may influence the effectiveness of this communication, or even whether this information is communicated at all. Pre- and in-service health worker training would be an important first step in closing this gap. In this study, we found that 13.7% of women whose babies were weighed did not know the birthweight. Whilst some of these women may have forgotten the weight, our qualitative findings provide evidence of communication challenges between healthcare providers and women with regard to birthweight, which are important to close.

Health cards are potentially an effective way of communicating information to a woman from one health provider to another, or to an interviewer in a household survey [31]. Health cards are therefore an important potential method for improving birthweight data in surveys. In this study, 62.4% of women reported that their baby was weighed at birth, but only a third of these had a health card recording this information available at the time of the survey. These findings are consistent with previous findings that even in settings where there is a policy for hand-held records, their practical utility is limited by factors such as lack of government funding to maintain implementation, regular stock outs and low quality of completion of various elements, including missing data and illegible entries [32, 33]. Our qualitative findings suggest that whilst health cards were seen as an important way to communicate birthweight, frequent challenges were faced including missing cards, cards being held by men and illegible or missing birthweight information.

Misreporting of birthweight is thought to be common in surveys. For half of babies reported to be weighed at birth, only information from recall and not card birthweight was available. Findings regarding the reliability of recalled birthweight data collected during routine surveys to adequately classify LBW babies have been varied [10, 34–38]. Errors due to heaping are especially pronounced in populations with higher LBW rates where a larger number of babies have a birthweight around the 2500-g cut-off. Overall, evidence suggests some errors in the precision of recalled birthweight at an individual level [2, 12]; however, no previous studies have sought to describe factors associated with increased heaping. In our study, heaping was not associated with socioeconomic status, but was less common in women with secondary or higher education compared to those with primary only or no education. Whilst heaping of birthweights at 500-g intervals was more marked in our recall data, consistent with other studies it was also evident in card data [12], suggesting that at least part of the issue may be due to errors in measurement or recording at the time of birth. Addressing these will require appropriately calibrated weighing scales and trained personnel with sufficient time to record results rapidly after measuring.

Our analysis showed a large gap between the proportion of facility livebirths and known birthweight, especially amongst neonatal deaths which varied by site. Closing the birthweight measurement gap for facility births is an important first step to improving the availability of birthweight data at a population level. In particular, attention should be given to improving the weighing of small and sick babies at birth and the communication of this information to their mothers. Health cards may have an important role to play in bridging gaps in the flow and availability of information, but only if health workers record the birthweight on these, even if the baby has died.

Strengths of this study include the large survey dataset from five LMICs, with consistent questions and analyses, plus multi-site comparable, qualitative data. To our knowledge, this is the first study to undertake qualitative research with both women and interviewers alongside a quantitative survey to seek to understand the reporting and measurement of birthweight. Since our study was undertaken with women in HDSS sites who were under regular surveillance, their knowledge about birthweight may differ from women not under surveillance. Our findings differ from a previous study in rural Bangladesh where knowing birthweight was not considered a priority [39]. In this study, 62% of babies were weighed compared to 48% across all DHS surveys between 2000 and 2016, and 21% compared to 14% had birthweight recorded on a card that was available at the time of the survey. However, in view of the large inter-site variations observed, and as some of these differences may be accounted for by increases in coverage of both facility birth and health cards over this time, it is likely that our results may be relevant across many LMICs. In this study, the assessment of birthweight data quality was limited by the women’s ability to report this information, an assessment of heaping and the plausibility of the distribution of reported birthweights. A further limitation was lack of information from health facilities to support the information and perceptions reported by women and interviewers in the qualitative data. However, a recent facility-based study in Tanzania found birthweight to be highly valued by health care providers, with barriers including gaps in weighing equipment and knowing how data would be used, thus aligning with our findings [29].

Hand-held records, either traditional paper-based health cards or electronic records could play an important role in closing this gap, but investments are required to improve understanding of how to ameliorate the quality of recorded information. Training interviewers in how best to use these card data to supplement women’s responses could facilitate more information being captured in household surveys. Further research is needed to explore challenges faced by health care providers in the communication of birthweight and other health-related information to women. This could include exploring how documentation can be streamlined to reduce duplication, strengthen the woman-provider relationship and enable women and health providers to inform the care that they receive and provide.

This paper has focused on improving birthweight capture and data quality in population-based surveys as an important short- to medium-term method to improve the availability of birthweight information at a population level. However, large-scale household surveys are expensive to undertake, with large time lags of up to 5 years between the birth and the capture of birthweight information and even longer before this information is available to inform policy and programmes. Investment in both HMIS and CRVS systems in many LMICs is increasing, and including birthweight information for every birth captured in these platforms as they expand will be important for improving access to timely data to drive action.

## Conclusions

Our study shows that whilst birthweight is perceived as valuable by women and communities, there are missed opportunities to improve birthweight data, even for facility births and especially for neonatal deaths. Even where birthweight is known, heaping of birthweight impacts utility for important public health indicators such as LBW.

Closing this gap will require investment in weighing every newborn, including those born at home and improving the quality of such measurements and the communication of this information to women, and within health systems. The potential of hand-held records to close this gap for women and to improve survey data has yet to be fully realised. Accurate birthweight data are needed for improving both individual clinical care for these most vulnerable babies and public health tracking to end preventable neonatal deaths and reduce long-term morbidity and disability in the SDG era.

## Supplementary information

**Additional file 1.** Background overview of the five HDSS sites.

**Additional file 2.** Selection of women with a livebirth surviving the neonatal period.

**Additional file 3.** STROBE guidelines checklist.

**Additional file 4.** Calculation of survey weights.

**Additional file 5.** Details of qualitative methods (FGDs), EN-INDEPTH study.

**Additional file 6:** Objective 1 - additional results on timing of questions. **Additional file 6.1:** Time taken to complete birthweight questions. **Additional file 6.2:** Distribution of time taken to complete perceived birthweight questions for the survey modules. **Additional file 6.3:** Distribution of time taken to complete actual birthweight questions for the survey modules.

**Additional file 7:** Objective 2 - additional results. **Additional file 7.1:** Characteristics of the live births included in the EN-INDE PTH birthweight analysis for the five sites weighted (*n* = 14710). **Additional file 7.2:** Factors associated with women’s report of baby being weighed at birth (*n* = 14,701). **Additional file 7.3:** Factors associated with being able to report birthweight for babies weighed at birth (n = 7,888). Additional file 7.4: Factors associated with availability of birthweight from card (*n* = 14,701). **Additional file 7.5:** Sensitivity analysis of mean birthweight by card and recall, excluding Matlab site. **Additional file 7.6:** Comparison of mean birthweight and proportion low birthweight by sex and survival status. **Additional file 7.7:** Factors associated with having a birthweight heaped on 500 g intervals (*n* = 7,888).

**Additional file 8:** Objective 3 - additional results. **Additional file 8.1:** Overview of perceived barriers and enablers to birthweight measurement in five HDSS (Women only). **Additional file 8.2** Overview of perceived barriers and enablers to reporting birthweight in household surveys in five HDSS.

**Additional file 9.** Ethical approval of local Institutional Review Boards.

## Data Availability

Data sharing and transfer agreements were jointly developed and signed by all collaborating partners. The datasets generated during the current study are deposited online at 10.17037/DATA.00001556 with data access subject to approval by collaborating parties.

## Abbreviations

DHS: Demographic and Health Surveys
ENAP: Every Newborn Action Plan
EN-INDEPTH: Every Newborn-International Network for the Demographic Evaluation of Populations and their Health
FBH+: Full birth history (+ denotes additional questions on pregnancy losses)
FGD: Focus group discussion
FPH: Full pregnancy history
HDSS: Health and demographic surveillance system
LMICs: Low- and middle-income countries
MICS: Multiple Indicator Cluster Survey
SDG: Sustainable Development Goals
UN: United Nations
WHO: World Health Organization
