# Supplementary material for: Birthweight data completeness and quality in population-based surveys: EN-INDEPTH study
Source: Popul Health Metr. 2021 Feb 8;19(Suppl 1):17. doi: 10.1186/s12963-020-00229-w (PMC7869202; doi:10.1186/s12963-020-00229-w)
Supplement: Supplementary file 1 — Additional file 1. Background overview of the five HDSS sites. [file 12963_2020_229_MOESM1_ESM.docx]

## Additional file 1: Background overview of the five HDSS sites

|  | **Bandim** | **Dabat** | **IgangaMayuge** | **Matlab** | **Kintampo** |
| --- | --- | --- | --- | --- | --- |
| **Country** | Guinea-Bissau | Ethiopia | Uganda | Bangladesh | Ghana |
| **HDSS start year** | 1978 | 1996 | 2004 | 1966 | 1994 |
| **Location** | Guinea-Bissau, covering rural and urban | Gondor, Amhara regional state, 821 km northwest of Addis Ababa and 75km north | Iganga and Mayuge districts, approximately, 120km east of capital, Kampala along Kenya-Uganda highway | Matlab Upazila, in Chandpur district, 55km southeast of capital, Dhaka | Brong Ahafo region |
| **Population** | 180,000 | 69,468 | 83,000 | 230,185 | 152,519 |
| **Households** | 36,000 | 16,016 | 16,000 | 53,226 | 32,000 |
| **Total Fertility Rate** | 4.3 | 3.8 | 4.3 | 2.6 | 4.1 |
| **Frequency of rounds** | Urban: Monthly Rural: 2/year | 2/year | 2/year | 6/year | 1/year |
| **Informants/**  **scouts** | Recently started using community key informants in parts of rural area | Local guides report within 48 hours | 64 Community based “scouts”  and VHTs | No | Community key informants |
| **Incentives for reporting** | Each woman asked pregnancy status and pregnancy ID given to pregnancy | 83% female enumerators | Informants and scouts are community members who are elected and accepted by the communities | Urine test; 100% female enumerators | Each woman asked pregnancy status |
| **Frequency of re-census the area** | Urban area every 2-7 years Rural: Every round | Every 7 years | Each update round | 8 years or more | Last census 2003 |
| **Facility births** | Urban: 65%  Rural: 39% | 17% | 64% | 69% (Intervention area: 87%; Comparison Area: 50%) | 61% |
| **Links to facility** | In national hospital, not in rural | Pilot study ongoing | No | Matlab hospital only (17% of births) | No |
| **Data entry** | Tablet planned in rural in 2020 | Piloting tablet | Paper-based | Galaxy Tab | Paper-based |
| **Birthweight** | Yes - Urban only | Yes – kg and maternal perception | Yes | Yes | Yes – Since 2015 |
| **GA** | Yes – at pregnancy registration | Yes | Yes | Yes | Yes |
| **Birth certificate** | Not routinely - On a subset around 400 women | No | No | Yes | No |
| **Death certificate** | Yes – asked with verbal autopsy | No | Yes – asked with verbal autopsy | Yes | Yes – asked with verbal autopsy |
| **Miscarriages/Termination of pregnancy (TOP)** | Yes - Miscarriages only | Yes - TOP differentiated from miscarriages | Yes - Miscarriages only | Yes - TOP differentiated from miscarriages | Yes - TOP differentiated from miscarriages |
