# Supplementary material for: Birthweight data completeness and quality in population-based surveys: EN-INDEPTH study
Source: Popul Health Metr. 2021 Feb 8;19(Suppl 1):17. doi: 10.1186/s12963-020-00229-w (PMC7869202; doi:10.1186/s12963-020-00229-w)
Supplement: Supplementary file 2 — Additional file 2. Selection of women with a livebirth surviving the neonatal period. [file 12963_2020_229_MOESM2_ESM.docx]

## Additional file 2: Selection of women with a livebirth surviving the neonatal period, EN-INDEPTH survey

Piloting of the EN-INDEPTH survey in the Survey Solutions app found that the full questionnaire took a long time. In order to reduce the overall time, whilst still reaching the target sample size for the main randomised comparison between FBH+ and FPH, a subset of women was selected to receive the pregnancy and birth module in their questionnaire (which is the longest module), only information regarding the most recent birth in each category was collected.

In view of the app set up, it was not possible to select women from the FPH arm for this part of the survey; therefore, only women who were in the FBH+ were eligible for selection. Selection was made at random by the app, with the initial intention that 30% of all women interviewed in the FBH+ arm who had a livebirth surviving the neonatal period (i.e. that did not result in a neonatal death) since 1^st^ January 2012 would be selected to receive the pregnancy and birth questionnaire module.

Women were sampled in slightly different ways across the sites. In Dabat, all women interviewed in the FBH+ with an eligible livebirth were selected to receive the pregnancy and birth module. In Matlab and Bandim, 30% of women in the FBH+ with an eligible livebirth were randomly selected to receive the pregnancy and birth module. In IgangaMayuge and Kintampo, for the first 1 – 2 months of data collection, all women interviewed in the FBH+ with an eligible livebirth were selected to receive the pregnancy and birth module; for the rest of the data collection period, 30% of women in the FBH+ with an eligible livebirth were randomly selected to receive the pregnancy and birth module.

Overall 50% of women in the FBH+ arm with an eligible livebirth received the pregnancy and birth module. The proportion that completed the module is detailed by site, below:

|  | Number of women in FBH+ arm completing pregnancy and birth module | Percentage of eligible women in FBH+ arm completing pregnancy and birth module | Percentage contribution to total surviving livebirths by site |
| --- | --- | --- | --- |
| Bandim | 1,316 | 28.3% | 9.8% |
| Dabat | 3,357 | 99.4% | 24.9% |
| IgangaMaguye | 1,917 | 64.3% | 14.2% |
| Kintampo | 3,951 | 66.7% | 29.3% |
| Matlab | 2,936 | 29.6% | 21.8% |
| Total | 13,477 | 50.2% | 100.0% |
