# Supplementary material for: Birthweight data completeness and quality in population-based surveys: EN-INDEPTH study
Source: Popul Health Metr. 2021 Feb 8;19(Suppl 1):17. doi: 10.1186/s12963-020-00229-w (PMC7869202; doi:10.1186/s12963-020-00229-w)
Supplement: Supplementary file 4 — Additional file 4. Calculation of survey weights. [file 12963_2020_229_MOESM4_ESM.docx]

## Additional file 4: Calculation of survey weights

The analysis was restricted to women with livebirths (survivors or neonatal deaths) interviewed in the FBH+ arm.

***Step 1:*** We calculated the probability of a woman with a livebirth outcome (surviving livebirth or neonatal death) since 1^st^ January 2012 receiving the additional questions in the pregnancy and birth module. For a woman with a neonatal death since 1^st^ January 2012 the probability of being included was 1, as all women with a neonatal death received these additional questions.

For a woman with a livebirth surviving the neonatal period, the probability of receiving the additional questions varied by HDSS site (see Annex 1). The weight was calculated as:

$$\frac{100}{\% of women in FBH+ arm completing pregnancy and birth module}$$

For example, in Bandim, the weight was calculated as 100/28.3.

***Step 2:*** The weight for each individual pregnancy outcome was calculated as the inverse of the probability of the pregnancy outcome being selected for each record. For example, if a woman had 2 neonatal deaths after 2012, only the last neonatal could be included so the individual sampling weight would be 2/1.

***Step 3:*** The weights calculated under steps 1 and 2 were normalized. First the mean weight of all the selected records was calculated. Then the weights calculated in step 2 were divided by the mean weight to estimate new weights. The mean of new weights is 1.

###

**Weighted numbers of live births by HDSS sites**

|  | **HDSS sites** | | | | | **All sites** |
| --- | --- | --- | --- | --- | --- | --- |
|  | ***Bandim*** | ***Dabat*** | ***IgangaMayuge*** | ***Kintampo*** | ***Matlab*** |  |
| Number of total live births in sub-sample (unweighted) | 1,559 | 3,486 | 2,045 | 4,416 | 3,185 | 14,411 |
| Number of total live births in sub-sample (weighted) | 2,518 | 1,801 | 1,658 | 3,220 | 5,225 | 14,411 |
| Number of live births surviving neonatal period in sub-sample (unweighted) | 1,316 | 3,356 | 1,917 | 3,951 | 2,936 | 13,476 |
| Number of live births surviving the neonatal period in sub-sample (weighted) | 2,469 | 1,880 | 1,913 | 3,454 | 4,318 | 14,034 |
| Number of neonatal deaths in sub-sample (unweighted) | 239 | 125 | 128 | 195 | 248 | 936 |
| Number of neonatal deaths in sub-sample (weighted) | 96 | 51 | 54 | 78 | 98 | 377 |
