# Supplementary material for: Birthweight data completeness and quality in population-based surveys: EN-INDEPTH study
Source: Popul Health Metr. 2021 Feb 8;19(Suppl 1):17. doi: 10.1186/s12963-020-00229-w (PMC7869202; doi:10.1186/s12963-020-00229-w)
Supplement: Supplementary file 5 — Additional file 5. Details of qualitative methods (FGDs), EN-INDEPTH study. [file 12963_2020_229_MOESM5_ESM.docx]

## Additional file 5: Details of qualitative methods (FGDs), EN-INDEPTH study

To identify community perceptions, practices, and barriers to reporting outcomes, and how these contribute to understanding of the measurement of any outcome in population-based surveys, 28 Focus Group Discussions (FGDs) were conducted with 82 EN-INDEPTH survey interviewers and supervisors and 172 women between February and August 2018. The number of FGDs undertaken was similar across the sites.

The FGDs explored women’s (respondents) and survey interviewers’ experiences with the EN-INDEPTH survey data collection process, as well as attitudes, knowledge, and practices around reporting and disclosure of pregnancy and adverse pregnancy outcomes and associated enablers and challenges.

A common training and implementation manual was developed for use across the sites. Interviews were held in the most commonly spoken local language (or English) by moderators and note takers who had skills in qualitative data collection methods. Data were transcribed locally and analysed by HDSS study teams, with at least two analysts participating in coding.

The following questions regarding birthweight were asked as part of the full EN-INDEPTH FGD interview guide available at: <https://doi.org/10.17037/DATA.00001556> ________________________________________________________________________________

**Woman’s FGD discussion guide**

**Part E: Gestational age and Birth weight**

In some cases when a woman is pregnant, babies are born too early or too small. We would like to learn from you a few things about this topic.

1. In this community do people think that it is important to count gestational age (from the first day of the woman's last menstrual cycle to the current date/ date when last pregnancy ended?)

- **Probe:** Why is it important? /Why is it not important? If it is important, is it easy for women to count this in this community? Why/ Why not?

1. In this community, do people think that weighing babies when they are born is important?

- **Probe:** Why is it important/ why is it not important? If it is important, how does a mother find out her baby’s birthweight in this community? Are there any problems with getting a baby weighed?
- **Probe:** What about if the baby is stillborn? Why is it important / why is it not important?

______________________________________________________________________________

**EN-INDEPTH survey interviewer’s discussion guide**

**Part C: Collecting data on pregnancy**

*Now, we would like to specifically talk about your experiences collecting data on pregnancies during the just concluded survey.*

1. What challenges did you face in collecting data on birth weight for the women’s children?

- **Probe:** difficulties for women in knowing the correct birth weight; why such difficulties; availability of health cards
