# Supplementary material for: Birthweight data completeness and quality in population-based surveys: EN-INDEPTH study
Source: Popul Health Metr. 2021 Feb 8;19(Suppl 1):17. doi: 10.1186/s12963-020-00229-w (PMC7869202; doi:10.1186/s12963-020-00229-w)

## Additional file 6: Objective 1- additional results on timing of questions

### Additional file 6.1: Time taken to complete birthweight questions

*mean time in minutes; **median time in minutes

|  | **Perceived Birthweight*** | | **Actual Birthweight** | |
| --- | --- | --- | --- | --- |
|  | **mean (SD)*** | **median (P25, P75)**** | **mean(SD)*** | **median (P25, P75) **** |
| **Post-neonatal survivors** | | | | |
| Overall | 0.4( 1) | 0.2( 0.1 ,0.4) | 0.2( 0.6) | 0.1( 0 ,0.1) |
| Survey Module |  |  |  |  |
| Full Birth History | 0.4( 1) | 0.2( 0.1 ,0.4) | 0.2( 0.6) | 0.1( 0 ,0.1) |
| Full Pregnancy History |  |  |  |  |
| HDSS |  |  |  |  |
| Bandim | 0.7( 1.4) | 0.3( 0.2 ,0.6) | 0.2( 0.4) | 0.1( 0.1 ,0.1) |
| Dabat | 0.2( 0.3) | 0.1( 0.1 ,0.2) | 0.1( 0.2) | 0.1( 0 ,0.1) |
| IgangaMayuge | 0.3( 0.6) | 0.2( 0.2 ,0.3) | 0.1( 0.3) | 0.1( 0 ,0.1) |
| Matlab | 0.4( 1.3) | 0.3( 0.2 ,0.4) | 0.2( 0.6) | 0.1( 0.1 ,0.1) |
| Kintampo | 0.5( 1.1) | 0.2( 0.1 ,0.4) | 0.2( 0.8) | 0.1( 0.1 ,0.2) |
| Birthweight method^ |  |  |  |  |
| Card | - | - | 0.2(0.9) | 0.1(0.0 ,0.1) |
| Recall | - | - | 0.1(0.5) | 0.1(0.0 ,0.1) |
| Don’t Know | - | - | 0.2(0.5) | 0.1(0.1 ,0.2) |
| **Neonatal deaths** | | | | |
| Overall | 0.5( 1.9) | 0.1( 0.1 ,0.3) | 0.4( 1.8) | 0.1( 0.1 ,0.2) |
| Survey Module |  |  |  |  |
| Full Birth History | 0.6( 2) | 0.1( 0.1 ,0.3) | 0.5( 1.9) | 0.1( 0.1 ,0.2) |
| Full Pregnancy History |  |  |  |  |
| HDSS |  |  |  |  |
| Bandim | 0.4( 1) | 0.2( 0.1 ,0.4) | 0.3( 1.1) | 0.1( 0.1 ,0.1) |
| Dabat | 0.4( 1.5) | 0.1( 0.1 ,0.2) | 0.4( 1.5) | 0.1( 0.1 ,0.2) |
| IgangaMayuge | 1.1( 3.6) | 0.1( 0.1 ,0.3) | 0.9( 3.4) | 0.1( 0.1 ,0.1) |
| Matlab | 0.6( 1.9) | 0.2( 0.1 ,0.3) | 0.4( 1.7) | 0.1( 0.1 ,0.2) |
| Kintampo | 0.3( 1.2) | 0.1( 0.1 ,0.3) | 0.3( 1.2) | 0.1( 0.1 ,0.2) |
| **Stillbirths** | | | | |
| Overall | 0.5( 1.5) | 0.2( 0.1 ,0.4) | 0.3( 1.1) | 0.1( 0.1 ,0.2) |
| Survey Module | 0( 0) | 0( 0 ,0) | 0( 0) | 0( 0 ,0) |
| Full Birth History | 0.5( 1.8) | 0.2( 0.1 ,0.4) | 0.3( 1.3) | 0.1( 0.1 ,0.2) |
| Full Pregnancy History |  |  |  |  |
| HDSS |  |  |  |  |
| Bandim | 0.5( 0.9) | 0.3( 0.1 ,0.5) | 0.3( 0.8) | 0.1( 0.1 ,0.2) |
| Dabat | 0.3( 0.9) | 0.1( 0.1 ,0.2) | 0.3( 1.1) | 0.1( 0.1 ,0.2) |
| IgangaMayuge | 0.5( 2.3) | 0.2( 0.1 ,0.3) | 0.4( 2.3) | 0.1( 0.1 ,0.1) |
| Matlab | 0.7( 2) | 0.3( 0.2 ,0.5) | 0.3( 0.8) | 0.1( 0.1 ,0.2) |
| Kintampo | 0.3( 0.7) | 0.2( 0.1 ,0.4) | 0.2( 0.7) | 0.1( 0.1 ,0.2) |

*mean time in minutes; **median time in minutes ^limited to babies reported to be weighed with timing information. Results only shown for post-neonatal survivors (n= 7,438), due to paucity of availability of information (only 32 neonatal deaths and 9 stillbirths were reported to be weighed and had information on timing available)

### Additional file 6.2: Distribution of time taken to complete perceived birthweight questions for the survey modules


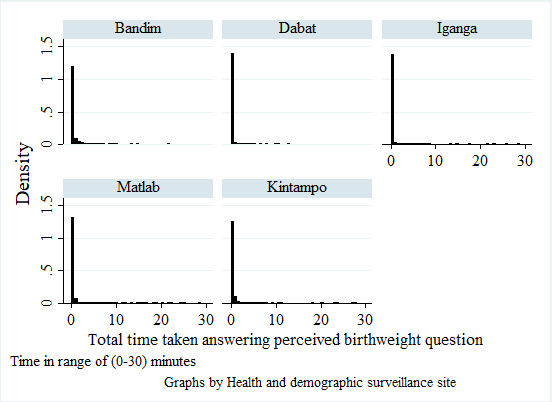


### Additional file 6.3: Distribution of time taken to complete actual birthweight questions for the survey modules


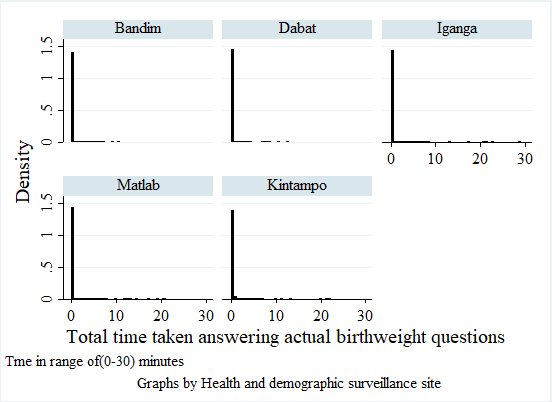

Supplement: Supplementary file 6 — Additional file 6: Objective 1 - additional results on timing of questions. Additional file 6.1: Time taken to complete birthweight questions. Additional file 6.2: Distribution of time taken to complete perceived birthweight questions for the survey modules. Additional file 6.3: Distribution of time taken to complete actual birthweight questions for the survey modules. [file 12963_2020_229_MOESM6_ESM.docx]
