# Supplementary material for: Birthweight data completeness and quality in population-based surveys: EN-INDEPTH study
Source: Popul Health Metr. 2021 Feb 8;19(Suppl 1):17. doi: 10.1186/s12963-020-00229-w (PMC7869202; doi:10.1186/s12963-020-00229-w)
Supplement: Supplementary file 7 — Additional file 7: Objective 2 - additional results. Additional file 7.1: Characteristics of the live births included in the EN-INDE PTH birthweight analysis for the five sites weighted (n = 14710). Additional file 7.2: Factors associated with women’s report of baby being weighed at birth (n = 14,701). Additional file 7.3: Factors associated with being able to report birthweight for babies weighed at birth (n = 7,888). Additional file 7.4: Factors associated with availability of birthweight from card (n = 14,701). Additional file 7.5: Sensitivity analysis of mean birthweight by card and recall, excluding Matlab site. Additional file 7.6: Comparison of mean birthweight and proportion low birthweight by sex and survival status. Additional file 7.7: Factors associated with having a birthweight heaped on 500 g intervals (n = 7,888). [file 12963_2020_229_MOESM7_ESM.docx]

## Additional file 7: Objective 2- additional results

### Additional file 7.1: Characteristics of the live births included in the EN-INDEPTH birthweight analysis for the five sites weighted (n=14,411)

|  | Bandim  n (%) | Dabat  n (%) | Iganga Mayuge  n (%) | Matlab  n (%) | Kintampo  n (%) | Overall  n (%) |
| --- | --- | --- | --- | --- | --- | --- |
| **Overall** | 2565 (17.8) | 1931 (13.4) | 1968 (13.7) | 4416 (30.6) | 3532 (24.5) | 14411 (100) |
| **Child’s characteristics** | | | | | | |
| **Sex** | | | | | | |
| Female | 1260 (49.1) | 992 (51.4) | 1004 (51.0) | 2153 (48.8) | 1752 (49.6) | 7161 (49.7) |
| Male | 1305 (50.9) | 939 (48.6) | 964 (49.0) | 2263 (51.2) | 1780 (50.4) | 7250 (50.3) |
| **Vital status** | | | | | | |
| Neonatal Death | 96 (3.7) | 51 (2.6) | 54 (2.8) | 98 (2.2) | 78 (2.2) | 377 (2.6) |
| Post-neonatal survivor | 2469 (96.3) | 1880 (97.4) | 1913 (97.2) | 4318 (97.8) | 3454 (97.8) | 14034 (96.7) |
| **Mother’s characteristics** | | | | | | |
| **Age** | | | | | | |
| 15 – 19 | 104 (4.1) | 53 (2.8) | 80 (4.0) | 203 (4.6) | 65 (1.8) | 504 (3.5) |
| 20 – 24 | 602 (23.5) | 320 (16.6) | 418 (21.3) | 1258 (28.5) | 494 (14.0) | 3093 (21.5) |
| 25 – 29 | 713 (27.8) | 501 (25.9) | 548 (27.9) | 1454 (32.9) | 808 (22.9) | 4024 (27.9) |
| 30 – 34 | 541 (21.1) | 430 (22.3) | 421 (21.4) | 926 (21.0) | 864 (24.5) | 3182 (22.1) |
| 35+ | 605 (23.6) | 626 (32.4) | 500 (25.4) | 575 (13.0) | 1300 (36.8) | 3606 (25.0) |
| **Education level** | | | | | | |
| No education | 901 (35.0) | 1257 (65.1) | 167 (8.5) | 161 (36.4) | 14745(41.2) | 3960 (27.5) |
| Primary | 703 (27.4) | 416 (21.6) | 1085 (55.1) | 776 (17.6) | 1529 (43.3) | 4509 (31.3) |
| Secondary | 806 (31.4) | 120 (6.2) | 625 (31.8) | 2895 (65.5) | 487 (13.8) | 4932 (34.2) |
| Higher | 155 (6.1) | 138 (7.1) | 91 (4.6) | 585 (13.2) | 41 (1.2) | 1010 (7.0) |
| **Socioeconomic status** | | | | | | |
| Poorest | 484 (18.9) | 559 (29.0) | 528 (26.9) | 880 (19.9) | 698 (19.8) | 3149 (21.9) |
| 2 | 480 (18.7) | 373 (19.3) | 451 (22.9) | 881 (20.0) | 700 (19.8) | 2885 (20.0) |
| 3 | 445 (17.3) | 427 (22.1) | 366 (18.6) | 877 (19.9) | 698 (19.8) | 2813 (19.5) |
| 4 | 500 (19.5) | 321 (16.6) | 354 (18.0) | 932 (21.1) | 735 (20.8) | 2842 (19.7) |
| Richest | 656 (25.6) | 251 (13.0) | 268 (13.6) | 847 (19.2) | 701 (19.8) | 2722 (18.9) |
| **Religion** | | | | | | |
| Christian | 876 (34.1) | 1871 (96.9) | 815 (41.4) | 0 (0.0) | 2092 (59.2) | 5653 (39.2) |
| Muslim | 1115 (43.5) | 60 (3.1) | 1151 (58.5) | 3935 (89.1) | 1226 (34.7) | 7487 (52.0) |
| Other or none | 574 (22.4) | 0 (0.0) | 1 (0.0) | 481 (10.9) | 214 (6.1) | 1270 (8.2) |
| **Parity** | | | | | | |
| 1 | 454 (17.7) | 224 (11.1) | 220 (11.2) | 1159 (26.3) | 365 (10.4) | 2413 (16.7) |
| 2 | 616 (24.0) | 312 (16.2) | 330 (16.8) | 1748 (39.6) | 647 (18.3) | 3653 (25.4) |
| 3 | 509 (19.8) | 297 (15.4) | 277 (14.1) | 1031 (23.3) | 624 (17.7) | 2738 (19.0) |
| 4 | 329 (12.8) | 283 (14.7) | 278 (14.1) | 327 (7.4) | 580 (16.4) | 1798 (12.5) |
| 5+ | 657 (25.6) | 825 (42.7) | 861 (43.8) | 151 (3.4) | 1316 (37.3) | 3810 (26.4) |
| **Birth characteristic** | | | | | | |
| **Place of birth** | | | | | | |
| Facility | 1720 (68.9) | 763 (39.7) | 1725 (89.9) | 2187 (50.0) | 2362 (66.9) | 8756 (61.5) |
| Home | 760 (30.5) | 1138 (59.2) | 174 (9.1) | 1328 (30.3) | 1126 (31.9) | 4526 (31.8) |
| Other | 17 (0.7) | 22 (1.2) | 19 (1.0) | 862 (19.7) | 44 (1.2) | 993 (6.7) |

Population is babies who have birthweight questions asked, their characteristics and those of their mothers. Based on weighted analysis

### Additional file 7.2: Factors associated with women’s report of baby being weighed at birth (n=14,411)

|  | Crude OR | 95% Confidence Interval | | p-value | Adjusted OR^1^ | 95% Confidence Interval | | p-value |
| --- | --- | --- | --- | --- | --- | --- | --- | --- |
| **Site** |  |  |  |  |  |  |  |  |
| Bandim | 1.00 |  |  |  | 1.00 |  |  |  |
| Dabat | 0.14 | 0.12 | 0.16 | <0.001 | 0.12 | 0.09 | 0.15 | <0.001 |
| IgangaMayuge | 2.18 | 1.81 | 2.63 | <0.001 | 0.86 | 0.65 | 1.15 | 0320 |
| Matlab | 0.99 | 0.85 | 1.14 | 0.852 | 0.64 | 0.50 | 0.82 | 0.001 |
| Kintampo | 0.45 | 0.55 | 0.74 | <0.001 | 0.49 | 0.38 | 0.61 | <0.001 |
| **Birthplace** |  |  |  |  |  |  |  |  |
| Facility | 1.00 |  |  |  | 1.00 |  |  |  |
| A home | 0.02 | 0.02 | 0.03 | <0.001 | 0.03 | 0.02 | 0.03 | <0.001 |
| Other | 1.37 | 1.26 | 2.10 | <0.001 | 1.21 | 0.91 | 1.61 | 0.183 |
| **Vital Status** |  |  |  |  |  |  |  |  |
| Post-neonatal survivor | 1.00 |  |  |  | 1.00 |  |  |  |
| Neonatal death | 0.43 | 0.37 | 0.49 | <0.001 | 0.19 | 0.16 | 0.24 | <0.001 |
| **Maternal Age** |  |  |  |  |  |  |  |  |
| 15-19 | 1.00 |  |  |  | 1.00 |  |  |  |
| 20-24 | 0.92 | 0.75 | 1.13 | 0.419 | 1.11 | 0.81 | 1.47 | 0.552 |
| 25-29 | 0.92 | 0.75 | 1.13 | 0.437 | 1.29 | 0.95 | 1.76 | 0.103 |
| 30-34 | 0.88 | 0.71 | 1.08 | 0.215 | 1.45 | 1.04 | 2.03 | 0.031 |
| 35+ | 0.67 | 0.55 | 0.82 | <0.001 | 1.33 | 0.93 | 1.90 | 0.114 |
| **Maternal Education** |  |  |  |  |  |  |  |  |
| No education | 1.00 |  |  |  | 1.00 |  |  |  |
| Primary only | 2.80 | 2.51 | 3.11 | <0.001 | 1.43 | 1.20 | 1.71 | <0.001 |
| Primary & secondary | 4.63 | 4.14 | 5.18 | <0.001 | 2.12 | 1.73 | 2.61 | <0.001 |
| Higher | 11.28 | 8.95 | 14.22 | <0.001 | 3.92 | 2.96 | 5.18 | <0.001 |
| **Socioeconomic status** |  |  |  |  |  |  |  |  |
| Poorest | 1.00 |  |  |  | 1.00 |  |  |  |
| 2 | 1.36 | 1.21 | 1.53 | <0.001 | 1.11 | 0.92 | 1.34 | 0.265 |
| 3 | 1.45 | 1.29 | 1.64 | <0.001 | 1.08 | 0.90 | 1.30 | 0.408 |
| 4 | 2.07 | 1.83 | 2.35 | <0.001 | 1.25 | 1.04 | 1.51 | 0.017 |
| Richest | 2.71 | 2.35 | 3.13 | <0.001 | 1.53 | 1.24 | 1.86 | <0.001 |
| **Parity** |  |  |  |  |  |  |  |  |
| 1 | 1.00 |  |  |  | 1.00 |  |  |  |
| 2 | 0.77 | 0.68 | 0.86 | <0.001 | 1.04 | 0.87 | 1.26 | 0.652 |
| 3 | 0.59 | 0.52 | 0.68 | <0.001 | 0.96 | 0.77 | 1.20 | 0.737 |
| 4 | 0.50 | 0.43 | 0.58 | <0.001 | 0.90 | 0.68 | 1.18 | 0.448 |
| 5+ | 0.38 | 0.34 | 0.43 | <0.001 | 0.91 | 0.69 | 1.20 | 0.494 |
| **Religion** |  |  |  |  |  |  |  |  |
| Christian | 1.00 |  |  |  | 1.00 |  |  |  |
| Muslim | 1.71 | 1.57 | 1.86 | <0.001 | 0.96 | 0.80 | 1.16 | 0.682 |
| Other or none | 1.31 | 1.10 | 1.54 | 0.001 | 1.24 | 0.93 | 1.67 | 0.146 |

^1^Includes 14,245 babies with complete data for all variables

### Additional file 7.3: Factors associated with being able to report birthweight for babies weighed at birth (n=8,993)

|  | Crude OR | 95% Confidence Interval | | p-value | Adjusted OR^1^ | 95% Confidence Interval | | p-value |
| --- | --- | --- | --- | --- | --- | --- | --- | --- |
| **Site** |  |  |  |  |  |  |  |  |
| Bandim | 1.00 |  |  |  | 1.00 |  |  |  |
| Dabat | 0.10 | 0.07 | 0.13 | <0.001 | 0.06 | 0.04 | 0.08 | <0.001 |
| IgangaMayuge | 10.56 | 4.64 | 24.03 | <0.001 | 9.25 | 4.02 | 21.24 | <0.001 |
| Matlab | 0.77 | 0.58 | 1.03 | 0.073 | 0.58 | 0.42 | 0.79 | 0.001 |
| Kintampo | 0.24 | 0.18 | 0.31 | <0.001 | 0.22 | 0.16 | 0.30 | <0.001 |
| **Birthplace** |  |  |  |  |  |  |  |  |
| Facility | 1.00 |  |  |  | 1.00 |  |  |  |
| A home | 0.42 | 0.33 | 0.54 | <0.001 | 0.44 | 0.33 | 0.58 | <0.001 |
| Other | 0.96 | 0.74 | 1.25 | 0.775 | 0.66 | 0.48 | 0.90 | 0.010 |
| **Vital Status** |  |  |  |  |  |  |  |  |
| Post-neonatal survivor | 1.00 |  |  |  | 1.00 |  |  |  |
| Neonatal death | 0.45 | 0.35 | 0.57 | <0.001 | 0.30 | 0.22 | 0.41 | <0.001 |
| **Maternal Age** |  |  |  |  |  |  |  |  |
| 15-19 | 1.00 |  |  |  | 1.00 |  |  |  |
| 20-24 | 0.64 | 0.43 | 0.96 | 0.029 | 0.66 | 0.42 | 1.03 | 0.069 |
| 25-29 | 0.56 | 0.38 | 0.83 | 0.004 | 0.64 | 0.41 | 1.02 | 0.061 |
| 30-34 | 0.58 | 0.39 | 0.87 | 0.009 | 0.84 | 0.52 | 1.38 | 0.498 |
| 35+ | 0.35 | 0.24 | 0.52 | <0.001 | 0.63 | 0.38 | 1.04 | 0.070 |
| **Maternal Education** |  |  |  |  |  |  |  |  |
| No education | 1.00 |  |  |  | 1.00 |  |  |  |
| Primary only | 2.10 | 1.75 | 2.52 | <0.001 | 1.30 | 1.05 | 1.62 | 0.016 |
| Primary & secondary | 3.89 | 3.22 | 4.71 | <0.001 | 2.07 | 1.60 | 2.67 | <0.001 |
| Higher | 7.18 | 4.89 | 10.55 | <0.001 | 5.25 | 3.27 | 8.43 | <0.001 |
| **Socioeconomic status** |  |  |  |  |  |  |  |  |
| Poorest | 1.00 |  |  |  | 1.00 |  |  |  |
| 2 | 1.10 | 0.86 | 1.40 | 0.440 | 1.34 | 1.04 | 1.80 | 0.024 |
| 3 | 0.99 | 0.78 | 1.25 | 0.938 | 1.31 | 1.00 | 1.72 | 0.046 |
| 4 | 0.95 | 0.76 | 1.20 | 0.677 | 1.33 | 1.01 | 1.73 | 0.039 |
| Richest | 1.03 | 0.82 | 1.29 | 0.809 | 1.48 | 1.13 | 1.95 | 0.004 |
| **Parity** |  |  |  |  |  |  |  |  |
| 1 | 1.00 |  |  |  | 1.00 |  |  |  |
| 2 | 0.90 | 0.73 | 1.12 | 0.346 | 1.07 | 0.83 | 1.37 | 0.620 |
| 3 | 0.77 | 0.62 | 0.97 | 0.025 | 1.03 | 0.77 | 1.40 | 0.823 |
| 4 | 0.65 | 0.50 | 0.83 | 0.001 | 1.03 | 0.73 | 1.45 | 0.872 |
| 5+ | 0.51 | 0.42 | 0.63 | <0.001 | 0.89 | 0.63 | 1.28 | 0.542 |
| **Religion** |  |  |  |  |  |  |  |  |
| Christian | 1.00 |  |  |  | 1.00 |  |  |  |
| Muslim | 1.68 | 1.45 | 1.95 | <0.001 | 0.80 | 0.64 | 1.00 | 0.045 |
| Other or none | 1.11 | 0.85 | 1.45 | 0.457 | 0.65 | 0.46 | 0.91 | 0.012 |

^1^Includes weighted count 8,881 babies with complete data for all variables

### Additional file 7.4: Factors associated with availability of birthweight from card (n=14,411)

|  | Crude OR | 95% Confidence  Interval | | p-value | Adjusted OR^1^ | 95% Confidence  Interval | | p-value |
| --- | --- | --- | --- | --- | --- | --- | --- | --- |
| **Site** |  |  |  |  |  |  |  |  |
| Bandim | 1.00 |  |  |  | 1.00 |  |  |  |
| Dabat | 0.02 | 0.01 | 0.02 | <0.001 | 0.02 | 0.02 | 0.03 | <0.001 |
| IgangaMayuge | 0.35 | 0.29 | 0.41 | <0.001 | 0.16 | 0.13 | 0.20 | <0.001 |
| Matlab | 0.01 | 0.01 | 0.02 | <0.001 | 0.01 | 0.00 | 0.01 | <0.001 |
| Kintampo | 0.70 | 0.61 | 0.81 | <0.001 | 0.69 | 0.58 | 0.83 | <0.001 |
| **Birthplace** |  |  |  |  |  |  |  |  |
| Facility | 1.00 |  |  |  | 1.00 |  |  |  |
| A home | 0.07 | 0.06 | 0.09 | <0.001 | 0.06 | 0.05 | 0.08 | <0.001 |
| Other | 0.09 | 0.05 | 0.13 | <0.001 | 0.53 | 0.31 | 0.93 | 0.026 |
| **Vital Status** |  |  |  |  |  |  |  |  |
| Post-neonatal survivor | 1.00 |  |  |  | 1.00 |  |  |  |
| Neonatal death | 0.15 | 0.11 | 0.21 | <0.001 | 0.07 | 0.05 | 0.10 | <0.001 |
| **Maternal Age** |  |  |  |  |  |  |  |  |
| 15-19 | 1.00 |  |  |  | 1.00 |  |  |  |
| 20-24 | 0.88 | 0.69 | 1.15 | 0.341 | 0.89 | 0.63 | 1.28 | 0.541 |
| 25-29 | 0.92 | 0.71 | 1.20 | 0.537 | 0.91 | 0.63 | 1.32 | 0.629 |
| 30-34 | 1.20 | 0.92 | 1.56 | 0.184 | 1.14 | 0.77 | 1.69 | 0.522 |
| 35+ | 1.13 | 0.87 | 1.47 | 0.368 | 0.92 | 0.60 | 1.39 | 0.680 |
| **Maternal Education** |  |  |  |  |  |  |  |  |
| No education | 1.00 |  |  |  | 1.00 |  |  |  |
| Primary only | 1.42 | 1.25 | 1.63 | <0.001 | 1.26 | 1.05 | 1.50 | 0.013 |
| Primary & secondary | 0.89 | 0.78 | 1.02 | 0.103 | 1.49 | 1.21 | 1.84 | <0.001 |
| Higher | 0.68 | 0.53 | 0.87 | 0.002 | 1.32 | 0.90 | 1.92 | 0.158 |
| **Socioeconomic status** |  |  |  |  |  |  |  |  |
| Poorest | 1.00 |  |  |  | 1.00 |  |  |  |
| 2 | 1.21 | 1.03 | 1.41 | 0.021 | 1.03 | 0.85 | 1.26 | 0.741 |
| 3 | 1.10 | 0.93 | 1.29 | 0.261 | 0.90 | 0.73 | 1.11 | 0.318 |
| 4 | 1.13 | 0.96 | 1.33 | 0.138 | 0.77 | 0.63 | 0.94 | 0.009 |
| Richest | 1.04 | 0.88 | 1.22 | 0.669 | 0.59 | 0.48 | 0.73 | <0.001 |
| **Parity** |  |  |  |  |  |  |  |  |
| 1 | 1.00 |  |  |  | 1.00 |  |  |  |
| 2 | 0.86 | 0.74 | 1.00 | 0.056 | 1.07 | 0.87 | 1.32 | 0.532 |
| 3 | 0.95 | 0.81 | 1.12 | 0.555 | 1.01 | 0.79 | 1.28 | 0.948 |
| 4 | 1.30 | 1.09 | 1.56 | 0.004 | 1.05 | 0.80 | 1.39 | 0.724 |
| 5+ | 1.15 | 0.99 | 1.33 | 0.059 | 0.94 | 0.71 | 1.25 | 0.659 |
| **Religion** |  |  |  |  |  |  |  |  |
| Christian | 1.00 |  |  |  | 1.00 |  |  |  |
| Muslim | 0.56 | 0.51 | 0.63 | <0.001 | 1.18 | 1.01 | 1.38 | 0.036 |
| Other or none | 0.77 | 0.63 | 0.94 | 0.010 | 1.22 | 0.91 | 1.64 | 0.174 |

^1^ Adjusted for all other variables shown in the table. Includes weighted count 14,245 with complete information for all variables.

### Additional file 7.5: Sensitivity analysis of mean birthweight by card and recall, excluding Matlab site

|  | Birthweight from recall | | | Birthweight from card | | |
| --- | --- | --- | --- | --- | --- | --- |
|  | Number of babies | Mean birthweight (kg) | Low birthweight  n (%) | Number of babies | Mean birthweight (kg) | Low birthweight  n (%) |
| **Overall** | 4702 | 3.01 (2.98-3.04) | 802 (17.1) | 3057 | 3.13 (3.10-3.16) | 274 (9.0) |
| **Four sites (Excluding Matlab)** | 2011 | 3.24 (3.20 – 3.29) | 213 (10.6) | 3014 | 3.14 (3.11 – 3.16) | 263 (8.7) |

### Additional file 7.6: Factors associated with having a birthweight heaped on 500g intervals (n=7,653)

|  | Adjusted OR^1^ | 95% Confidence Interval | | p-value |
| --- | --- | --- | --- | --- |
| **Site** |  |  |  |  |
| Bandim | 1.00 |  |  |  |
| Dabat | 14.25 | 10.13 | 20.03 | <0.001 |
| IgangaMayuge | 14.91 | 11.37 | 19.55 | <0.001 |
| Matlab | 5.23 | 3.91 | 7.00 | <0.001 |
| Kintampo | 2.19 | 1.72 | 2.80 | <0.001 |
| **Birthplace** |  |  |  |  |
| Facility | 1.00 |  |  |  |
| A home | 1.61 | 1.18 | 2.19 | 0.003 |
| Other | 0.62 | 0.49 | 0.78 | <0.001 |
| **Reporting Method** |  |  |  |  |
| KG FROM CARD | 1.00 |  |  |  |
| KG FROM RECALL | 2.59 | 2.11 | 3.19 | <0.001 |
| **Vital Status** |  |  |  |  |
| Post-neonatal survivor | 1.00 |  |  |  |
| Neonatal death | 1.32 | 0.97 | 1.81 | 0.080 |
| **Maternal Age** |  |  |  |  |
| 15-19 | 1.00 |  |  |  |
| 20-24 | 1.10 | 0.79 | 1.55 | 0.568 |
| 25-29 | 0.98 | 0.69 | 1.40 | 0.908 |
| 30-34 | 0.92 | 0.63 | 1.34 | 0.654 |
| 35+ | 0.94 | 0.63 | 1.40 | 0.765 |
| **Maternal Education** |  |  |  |  |
| No education | 1.00 |  |  |  |
| Primary only | 0.92 | 0.73 | 1.17 | 0.505 |
| Primary & secondary | 0.73 | 0.56 | 0.94 | 0.014 |
| Higher | 0.49 | 0.36 | 0.69 | <0.001 |
| **Socioeconomic status** |  |  |  |  |
| Poorest | 1.00 |  |  |  |
| 2 | 1.13 | 0.91 | 1.41 | 0.255 |
| 3 | 0.99 | 0.80 | 1.23 | 0.944 |
| 4 | 1.12 | 0.90 | 1.39 | 0.322 |
| Richest | 1.06 | 0.85 | 1.32 | 0.610 |
| **Parity** |  |  |  |  |
| 1 | 1.00 |  |  |  |
| 2 | 1.26 | 1.04 | 1.54 | 0.021 |
| 3 | 1.32 | 1.03 | 1.68 | 0.026 |
| 4 | 1.21 | 0.90 | 1.61 | 0.208 |
| 5+ | 1.35 | 0.99 | 1.84 | 0.054 |
| **Religion** |  |  |  |  |
| Christian | 1.00 |  |  |  |
| Muslim | 1.03 | 0.84 | 1.25 | 0.782 |
| Other or none | 1.15 | 0.85 | 1.56 | 0.366 |

^1^ Adjusted for all other variables shown in the table. Weighed analysis based on 7,653 weighed babies with complete data.
