# Supplementary material for: Birthweight data completeness and quality in population-based surveys: EN-INDEPTH study
Source: Popul Health Metr. 2021 Feb 8;19(Suppl 1):17. doi: 10.1186/s12963-020-00229-w (PMC7869202; doi:10.1186/s12963-020-00229-w)
Supplement: Supplementary file 8 — Additional file 8: Objective 3 - additional results. Additional file 8.1: Overview of perceived barriers and enablers to birthweight measurement in five HDSS (Women only). Additional file 8.2 Overview of perceived barriers and enablers to reporting birthweight in household surveys in five HDSS. [file 12963_2020_229_MOESM8_ESM.docx]

## Additional file 8: Objective 3- additional results

### Additional file 8.1: Overview of perceived barriers and enablers to birthweight measurement in five HDSS (Women only)

|  | **Bandim** | **Dabat** | **IgangaMayuge** | **Matlab** | **Kintampo** |  |
| --- | --- | --- | --- | --- | --- | --- |
| Barriers | | | | | | |
| Home births | ✓  Esp. in certain ethic groups.  Long distance to health post. | ✓  Many women still give birth at home  Women do not like their babies to be weighed at home as they think the child will be exposed to the elements (e.g. wind and sunlight) | ✓ | ✓  Midwives or health workers rarely come to the house and even if they do they don’t bring scales | ✓  Women unwilling to travel to health clinic for weighing  Even if travel to clinic health workers may not attend to you |  |
| Being born in a private clinic |  |  | ✓ |  |  |  |
| Facilities without baby scales |  |  |  |  | ✓  Mothers made to stand on adult scale with and without baby to calculate baby’s approximate weight |  |
| Child deceased or stillbirth | ✓  No perceived benefit in weighing a dead child (especially if at home) |  | ✓  No perceived benefit in weighing stillborn children | ✓  All perceived no benefit in weighing stillborn children.  For facility stillbirths - the nurses show attitude of negligence instead of measuring the weight of the baby as stillborn babies might bring down the reputation of the hospital. | ✓  No perceived benefit in weighing stillborn children or those who die shortly after birth |  |
| Social perceptions/ spiritual beliefs |  | ✓  Some mothers perceived that a child who has a good weight and attractive body will be attacked by the ‘evil eye’ and may get ill or die as a result |  | ✓  Some mothers do not want to weigh their baby as others knowing the baby is healthy could lead the ‘evil eye’ to harm the baby. |  |  |
| Enablers | | | | | | |
| Perceived value of birthweight for live births | ✓  Useful to provide baseline for monitoring child growth | ✓  Knowing birthweight can help women to seek newborn care services  Women liked babies to be weighed in health facilities | ✓  Useful to know if baby is normal or preterm and needs extra warmth  Provides baseline for monitoring child growth  Needed for registering for national IDs | ✓  Important to know if baby born healthy | ✓  Useful to know if baby is small so can feed more frequently  Can help see if something wrong with the child  Useful to provide baseline for monitoring child growth |  |
| Perceived value of birthweight for stillbirths | ×  None reported |  | ✓  Can help health workers known cause |  | ✓  Can help plan so you know what to eat in a subsequent pregnancy to avoid another stillbirth  Can help health workers know the cause of the stillbirth |  |

### Additional file 8.2: Overview of perceived barriers and enablers to reporting birthweight in household surveys in five HDSS

| Women only | Interviewers only | Both |
| --- | --- | --- |

|  | **Bandim** | **Dabat** | **IgangaMayuge** | **Matlab** | **Kintampo** |  |
| --- | --- | --- | --- | --- | --- | --- |
| Barriers |  |  |  |  |  |  |
| Mother not informed of the weight | ✓  Baby separated from mother soon after birth e.g. very sick or stillborn. Even if weighed mother not informed |  | ✓  Mother reported seeing the child weighed, but not being told the weight |  | ✓  Many women not told birthweight  Women find it difficult to ask health workers questions, including about birthweight |  |
| Health card not available | ✓  Health cards held by the men |  | Variable by interview gender: Female interviewers thought most had card. Male interviewers thought most didn’t, and that women didn’t care about the child’s health card and often lose them |  | ✓  Perceived by interviewers that some women do not look after their cards well and they get misplaced  Some women cannot afford to buy the health card |  |
| Birthweight not recorded on the health card or birthweight illegible |  |  | ✓  Especially in births in a referral hospital or government health centres and for births occurring over the weekend |  | ✓  Noted a large problem even for facility births  More commonly written on ANC card, child’s weighing card not usually available at time of birth and failure to transcribe weight from ANC to weighing card  Some birthweight records illegible |  |
| Interviewer’s look at the incorrect health card |  |  |  |  | ✓  Birthweight is recorded more consistently in ANC card, but many interviewers only ask for child’s health card |  |
| Social perceptions/ spiritual beliefs |  |  |  | ✓  Some mothers want to hide the weight of their baby because they believe that when a baby born with good health and other people know about the weight then ‘evil eye’ can cause harm to the health of the baby. |  |  |
| Enablers | | | | | | |
| Education level of the woman |  |  |  |  | ✓  More educated women can recall the birthweight even without the card. |  |
| Health cards |  | ✓  Some women bring health cards to show interviewers |  |  |  |  |
